# The microbiome of diabetic foot ulcers: a comparison of swab and tissue biopsy wound sampling techniques using 16S rRNA gene sequencing

**DOI:** 10.1186/s12866-020-01843-2

**Published:** 2020-06-16

**Authors:** J. Travis, M. Malone, H. Hu, A. Baten, K. Johani, F. Huygens, K. Vickery, K. Benkendorff

**Affiliations:** 1 School of Environment, Science and Engineering, Southern Cross University, Lismore, NSW Australia; 2 Limb Preservation and Wound Research Academic Unit, Western Sydney LHD, Liverpool, Sydney, NSW 2170 Australia; 3 Infectious Diseases and Microbiology, School of Medicine, Western Sydney University, Campbelltown Campus, Liverpool, Sydney, 2170 Australia; 4 Ingham Institute of Applied Medical Research, Liverpool, Sydney, NSW 2170 Australia; 5 Surgical Infection Research Group Faculty of Medicine and Health Sciences, Macquarie University, Sydney, Australia; 6 Agresearch, Grasslands Research Centre, Palmerston North, New Zealand; 7 Central Military Laboratories and Blood Bank, Prince Sultan Military Medical City, Riyadh, Saudi Arabia; 8 Institute of Health and Biomedical Innovation, Queensland University of Technology, Herston, QLD Australia; 9 School of Biomedical Science, Queensland University of Technology, Brisbane, Australia; 10 National Marine Science Centre, 2 Bay Drive, Coffs Harbour, NSW Australia

**Keywords:** Diabetic foot ulcers, Wound sampling methods, Swabs, Tissue biopsies, 16S rRNA gene sequencing, Wound pathogens

## Abstract

**Background:**

Health-care professionals need to collect wound samples to identify potential pathogens that contribute to wound infection. Obtaining appropriate samples from diabetic foot ulcers (DFUs) where there is a suspicion of infection is of high importance. Paired swabs and tissue biopsies were collected from DFUs and both sampling techniques were compared using 16S rRNA gene sequencing.

**Results:**

Mean bacterial abundance determined using quantitative polymerase chain reaction (qPCR) was significantly lower in tissue biopsies (*p* = 0.03). The mean number of reads across all samples was significantly higher in wound swabs $\Big(\overline{X}$ = 32,014) compared to tissue ($\overline{X}$ = 15,256, *p* = 0.001). Tissue biopsies exhibited greater overall diversity of bacteria relative to swabs (Shannon’s H diversity *p* = 0.009). However, based on a presence/absence analysis of all paired samples, the frequency of occurrence of bacteria from genera of known and potential pathogens was generally higher in wound swabs than tissue biopsies. Multivariate analysis identified significantly different bacterial communities in swabs compared to tissue (*p* = 0.001). There was minimal correlation between paired wound swabs and tissue biopsies in the number and types of microorganisms. RELATE analysis revealed low concordance between paired DFU swab and tissue biopsy samples (Rho = 0.043, *p* = 0.34).

**Conclusions:**

Using 16S rRNA gene sequencing this study identifies the potential for using less invasive swabs to recover high relative abundances of known and potential pathogen genera from DFUs when compared to the gold standard collection method of tissue biopsy.

## Background

Diabetic foot ulcers (DFUs) are associated with morbidity and mortality, and are a frequent cause of hospitalisation [1]. The majority of DFUs develop due to loss of protective sensation and/or reduced peripheral perfusion. Damage to the protective skin envelope enables entry of microorganisms which colonise host tissue. This may lead to further microbial replication and damage to host tissue which can manifest as a clinical infection [2]. Alternatively, the colonisation of microorganisms may lead to a stable microbial community, which does not elicit a host response, but contributes to several ill-defined mechanisms keeping the wound in a “chronic” non-healing state [3]. In either case, accurately identifying the microbiome within DFUs could enhance treatment by enabling targeted therapeutic regimens, which include the use of systemic or topical antimicrobial therapies in order to augment sharp debridement.

Historically, microorganisms associated with samples collected from all aspects of human disease have been identified in clinical and research laboratories using culture-dependent techniques, which employ traditional growth media and incubation conditions. These techniques are now acknowledged as being selective for microorganisms which thrive under the physiological and nutritional constraints of the microbiology laboratory [4]. Fastidious microorganisms are rarely isolated from culture, as they require specific growth conditions and identification techniques that are beyond the scope of most clinical laboratories. Therefore, many microorganisms that may be of clinical significance within wounds can remain uncultured and results may not accurately reflect the wound microbiome [5–7]. A review discussing classification, epidemiology and microbiology of skin and soft tissue infections, including diabetic foot ulcers, presented a summary of organisms isolated from 271 DFUs using culture techniques [8]. Percentages of common facultative aerobic isolates included *Staphylococcus* spp. (27%), *Pseudomonas* spp. (20%) and Enterobacteriaceae (11%). Anaerobes were isolated in 1.9% of the 271 wounds and the low percentages of anaerobes may be the result of a failure to isolate these organisms using traditional culture methods [9].

Next generation DNA sequencing, using variable regions of the bacterial 16S rRNA gene, has been successfully applied to provide an extended view of the chronic wound microbiome [4, 5] and more recently of infected tissues from DFUs [10]. The sampling techniques utilised in these studies have varied, with some researchers preferring wound swabs [11] rather than tissue biopsies or debridement material [10, 12]. The major limitation to obtaining tissue specimens in the form of a punch biopsy is that the procedure is more invasive, requires a skilled clinician, and in the absence of peripheral neuropathy, local anaesthesia. Furthermore, where study objectives require longitudinal sampling of wounds, obtaining tissue biopsies can present a challenge. Ethical implications must be considered, as regular sampling would be required for longitudinal studies, which could be distressing for participants, may affect wound healing and generally influence decisions to participate in the study.

In contrast, using a swab to obtain material from the surface of a wound is not invasive and does not involve any of the above sampling technique disadvantages. A number of research groups have routinely utilized wound swab samples for DNA sequencing studies in preference to tissue biopsies or curettage [4, 13, 14]. The limitations of collecting wound swabs has been investigated using cultivation-based studies which have reported low concordance of isolated bacteria when compared to tissue specimens [15, 16]. A large, multicentre study recruiting 400 participants with suspected infected diabetic foot ulcers from 25 sites within England investigated the concordance of cultured swabs collected using the Levine method [17] and tissue biopsies. Tissue sampling and culture methods were not standardised across laboratories. Overall there were significant differences in the pathogens reported from 395 paired tissue biopsies and swabs, with tissue biopsies isolating more pathogens than swabs [18–20]. This evidence base has therefore led many expert groups in the area of diabetic foot disease to promote the use of tissue biopsy or curettage as the gold standard sampling method for detecting the pathogens in infected DFUs [2, 21, 22]. Nevertheless, low concordance between sampling methods does not imply that one method is superior to the other, as the question still remains as to which method recovers a better representation of the wound microbiome and if swabs can be used to recover the majority of known and potential pathogen genera from DFUs.

Limited studies have been undertaken to directly compare concordance between microorganisms identified from wound swabs versus tissue biopsies using 16S rRNA gene sequencing. However, some researchers have utilised wound swab samples to produce robust, quality outcomes [4, 11, 23]. The microbiome of five samples from the inner elbow of healthy human skin tissue using tissue biopsies, skin scrapings and swabs was investigated [23] and authors found that the dominant microorganisms were identified equally well across the three collection methods. Conversely, other researchers have reported that tissue specimens reveal significantly more known and potential pathogen genera (*p* = 0.03) relative to those found in swabs [24].

These contrasting results provide the context as to why further research is required for understanding whether different sampling methods such as wound swabs or tissue biopsies produce comparable outcomes. With the increased utilisation of genomic approaches, including next generation DNA sequencing, there are clear logistical advantages to utilising wound swabs if the data is equivalent to, or just as informative as, tissue biopsies. The ability to collect samples quickly and easily with minimal training requirements could facilitate greater participant recruitment (because no contra-indications exist for collection of wound swabs when compared to tissue biopsies), and should enable temporal analysis on a larger scale. Therefore, the aim of this study was to investigate the concordance between paired swab and tissue samples from DFUs using 16S rRNA gene sequencing, and to determine if either or both sampling methods provide useful and clinically relevant information. Secondary aims were to compare information regarding microbial diversity and community structure of DFUs, knowledge of which may be insightful for researchers investigating therapeutic and diagnostic measures of these recalcitrant chronic wounds.

## Results

### Total bacterial abundance and number of reads

**Fig. 1:**
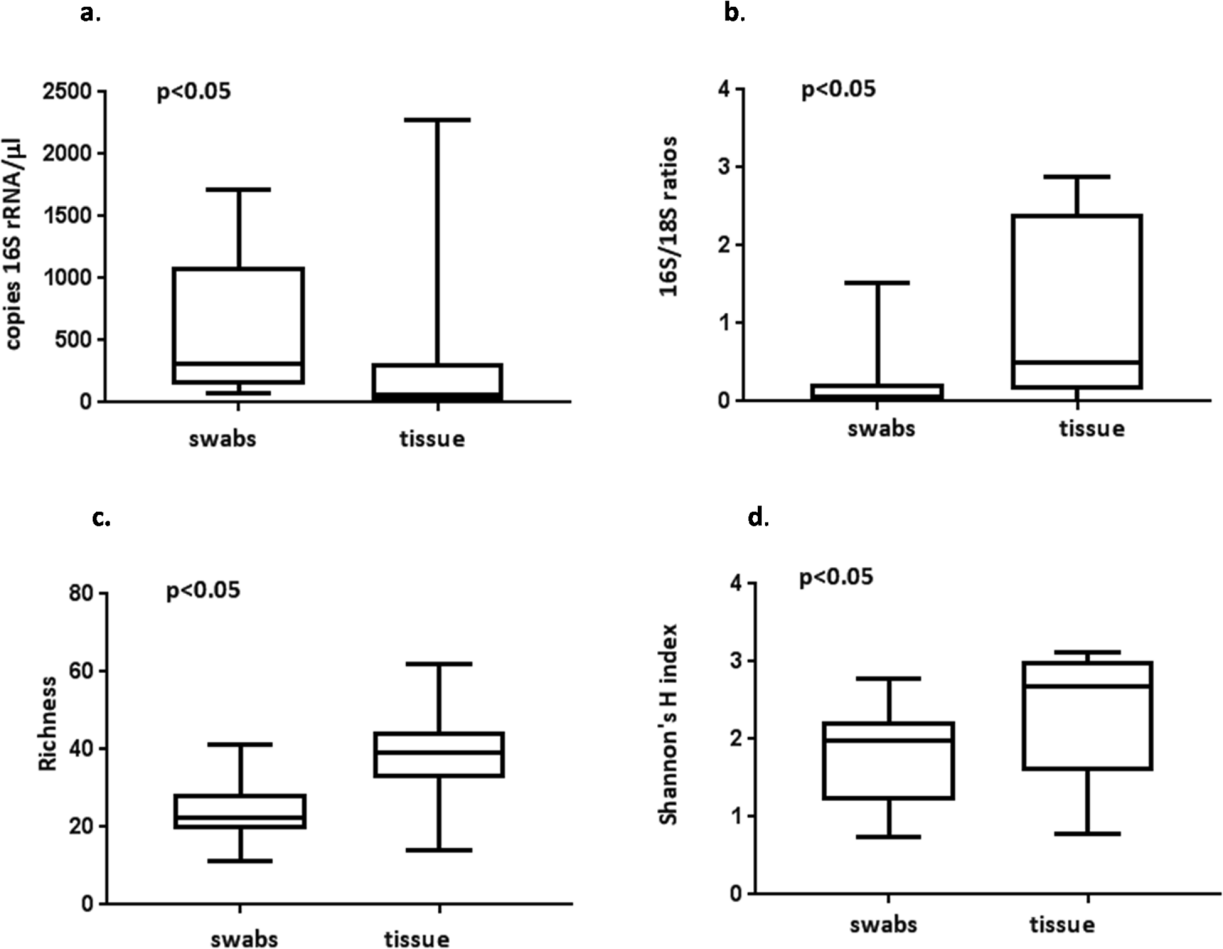
Bacterial abundance (qPCR) and sequence metrics for paired swab and tissue samples *N* = 20: **a**) number of copies of 16S rRNA gene /μl (sqrt transformed); **b**) swab and tissue biopsy 16S/18S ratios; **c**) richness (number of distinct OTUs) rarefied to 1500 reads; and **d**) standardised Shannon’s H index

The mean number of copies of the 16S rRNA gene/μl was significantly higher in swab than tissue biopsy samples (*p* = 0.03, Table 1). The range in total abundance was greater in tissue than swab samples (Fig. 1.a, Supplementary Table S1 Additional File 1). The ratios of the copies of 16S/18S rRNA gene indicated that tissue biopsies had a higher ratio of 16S rRNA gene copies relative to swabs with results of the Wilcoxin paired t-test significant (*p* = 0.004 Table 1, Fig. 1.b). The mean number of reads obtained from the swab samples was significantly higher than those from tissue samples (*p* = 0.001, Table 1). Rarefaction analysis established a suitable cut-off of 1500 reads for a standardised comparison and reads < 1500 were excluded from further analysis. No significant correlation was detected between swab and tissue biopsy samples for total abundance or number of reads, indicating no concordance in the order that swabs and tissue from the same DFU were ranked (Supplementary Table S2 Additional File 1).

**Table 1:** Summary of 16S rRNA gene sequencing analyses comparing paired swabs and tissue biopsies from 20 diabetic foot ulcerss

Parameter	Swabs Means (±s.d.)	Tissue Means (±s.d.)	w/t value^**1**^	***p*** value
No. of reads ^a^	32,014(±16,068)	15,256(±16,699)	− 162	0.001*
^d^Abundance ^b^	564.7(±566.9)	266.5(±535.5)	− 108	0.03*
16S/18S ratio	0.201(±0.35)	1.06(±1.08)	150	0.004*
Bacterial richness^c^	25.1 (±8.2)	38(±11.7)	4.423	0.0003*
Shannon’s diversity	1.8(±0.58)	2.4(±0.78)	2.921	0.009*
^d^*Staphylococcus* spp.	49.3(±58.2)	14.4(±19.3)	− 158	< 0.001*
^d^*Streptococcus* spp.	32.6(±39.3)	12.9(±37.9)	− 131	0.003*
^d^*Enterococcus* spp.	7.3 (±8.0)	9.2(±22.4)	− 57	0.224
^d^*Corynebacterium* spp.	42.9(±35.8)	18.3 (±20.0)	− 128	0.015*
^d^Enterobacteriaceae	49.3 (±49.1)	24.7(±35.5)	− 120	0.024*
^d^*Pseudomonas* spp.	11.8(±26.9)	16.6(±38.5)	8	0.776
^d^*Anaerococcus* spp.	39.1(±35.9)	17(±26.1)	− 132	0.012*
^d^*Finegoldia* spp.	20.8(±15.6)	17.8(±22.4)	−32	0.535

^1^ As most of the data analysed was nonparametric, Wilcoxin signed rank tests were used except for bacterial richness and Shannon’s diversity data which were subject to paired t-test analyses

^a^ reads in filtered OTU table (> 0.05%)

^b^ no. of copies of 16S rRNA gene/μl

^c^ Bacterial richness rarefied to 1500 reads

^d^ square root transformed data

* Significant results (*p* < 0.05)

### Species richness and diversity

Based on rarefaction for 1500 reads the bacterial richness derived from the number of distinct operational taxanomic units (OTUs), varied from a minimum of 11 and a maximum of 41 across all swab samples and from a minimum of 14 to a maximum of 62 across all tissue samples (Fig. 1.c, and Supplementary Table S3 Additional File 1). The mean rarefied bacterial richness (Table 1) was significantly higher (*p* = 0.0003) in tissue biopsy samples. The standardised species diversity (Shannon’s H index) was also higher in tissue (Fig. 1.d) and the paired t-test confirmed a significant difference in diversity (*p* = 0.009) between the two sampling techniques (Table 1).

### Common genera with known pathogens

**Fig. 2:**
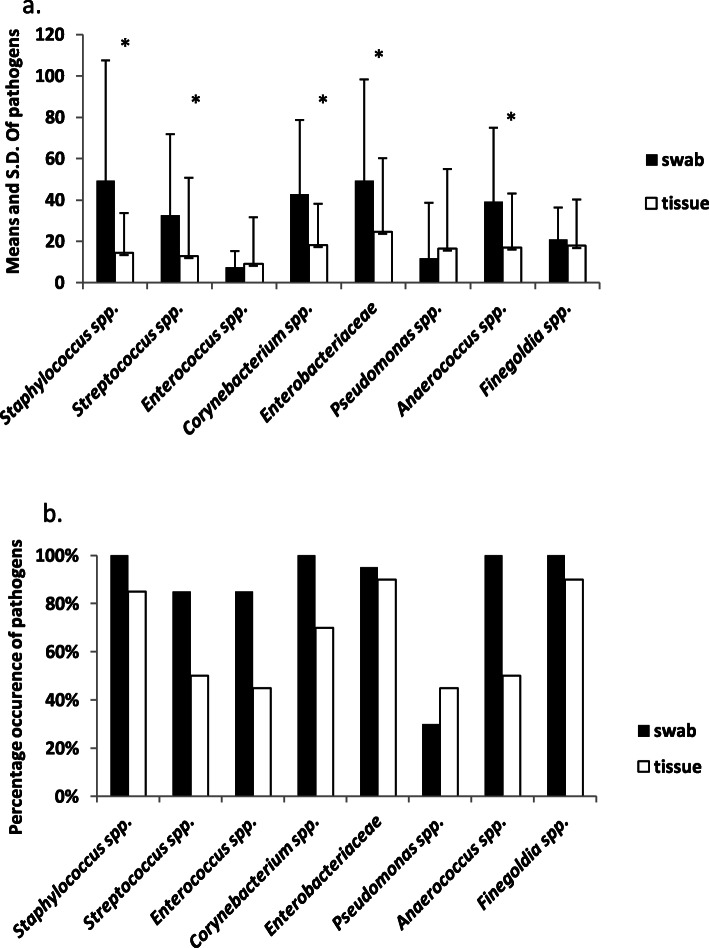
Comparison of common known and potential pathogen genera in swab and tissue biopsy samples: **a**) mean and standard deviation of square root transformed relative abundance of pathogens, * *p* < 0.05; and **b**) percent of swab and tissue samples in which each pathogen genera occurred

The mean relative abundance of bacteria from genera with known and potential pathogens was frequently higher in the swab samples compared to tissues (Table 1, Fig. 2.a) and this difference was significant for *Staphylococcus* spp.*, Streptococcus* spp.*, Corynebacterium spp.,* Enterobacteriaceae and the obligate anaerobe *Anaerococcus* spp. (*p* < 0.05, Table 1). In contrast, there were no significant differences in the mean relative abundances of *Enterococcus* spp.*, Pseudomonas* spp., and *Finegoldia* spp., in swabs and tissue samples. Results of the Spearman rank correlation indicate that none of the pathogens were significantly correlated in swabs and tissue from the same DFU (Supplementary Table S2 Additional File 1).

Based on presence or absence in each sample, the percentage of samples in which the genera of known species specific pathogens were detected is illustrated in Fig. 2.b. *Staphylococcus* spp., was found in all 20 swab samples, along with *Corynebacterium* spp.*, Anaerococcus* spp., and *Finegoldias* spp. By comparison, recovery of *Staphylococcus* spp., from the tissues samples was 85%, whereas 70% of tissue samples contained *Corynebacterium* spp.*,* 50% contained *Anaerococcus* spp., and 90% *Finegoldia* spp. Conversely, *Pseudomonas* spp. was identified in 45% of tissue samples compared to 30% of swab samples.

### Bacterial profile and additional organisms identified in swabs and tissue biopsies

**Fig. 3:**
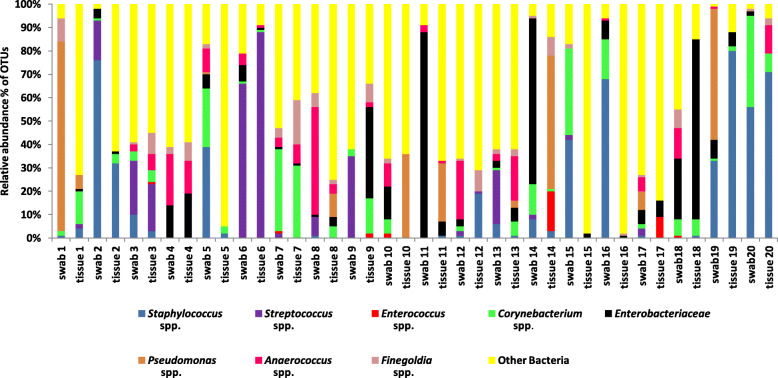
Relative abundance of common known and potential pathogen genera and other bacteria identified in each paired swab and tissue biopsy sample

Relative abundances of bacteria in genera containing known pathogens and other bacteria (Fig. 3) demonstrate very little concordance between swab and tissue sample pairs. Nevertheless, some samples do have similar populations of potential pathogens; for example both swab 3 and tissue 3 are dominated by *Streptococcus* spp., *Corynebacterium* spp., *Staphylococcus* spp., *Finegoldia* spp. and *Anaerococcus* spp. Paired swab and tissue samples from wound 4 recovered *Finegoldia* spp., *Anaerococcus* spp. and Enterobacteriaceae, and wound 6 samples have high abundances of *Streptococcus* spp. and very low abundances of Enterobacteriaceae, *Corynebacterium* spp. and *Anaerococcus* spp. However, other pairs are more variable with different bacteria in swabs and tissue, including wound swab sample 12, which had 25% abundance of *Anaerococcus* spp., but this organism was not identified in the tissue biopsy sample. On the other hand the tissue sample recovered 19% *Staphylococcus* spp., which was identified at an abundance of only 1% in the swab. There are many other examples of lack of concordance in the relative abundance of potential pathogens and other bacteria identified from the paired samples (Fig. 3).

Other bacterial genera (Fig. 3) and families identified from the samples used in this study have been listed (Supplementary Table S4 Additional File 2) with over 30% of the described organisms present in both swabs and tissue biopsies. The relative abundance of these additional organisms varied between samples (Fig. 3), and the majority of distinct OTUs were present at less than or equal to 1% of total OTUs in both swabs and tissue biopsies. Obligate anaerobes *Prevotella* spp., *Bacteroides* spp., *Porphyromonas* spp. and *Peptostreptococcus* spp. were present in relative abundances of up to 3% in tissue and 4% in swab samples (Supplementary Table S4 Additional File 2). Archaea were identified from tissue samples only, in relative abundances of up to 1%.

### Community composition

**Fig. 4:**
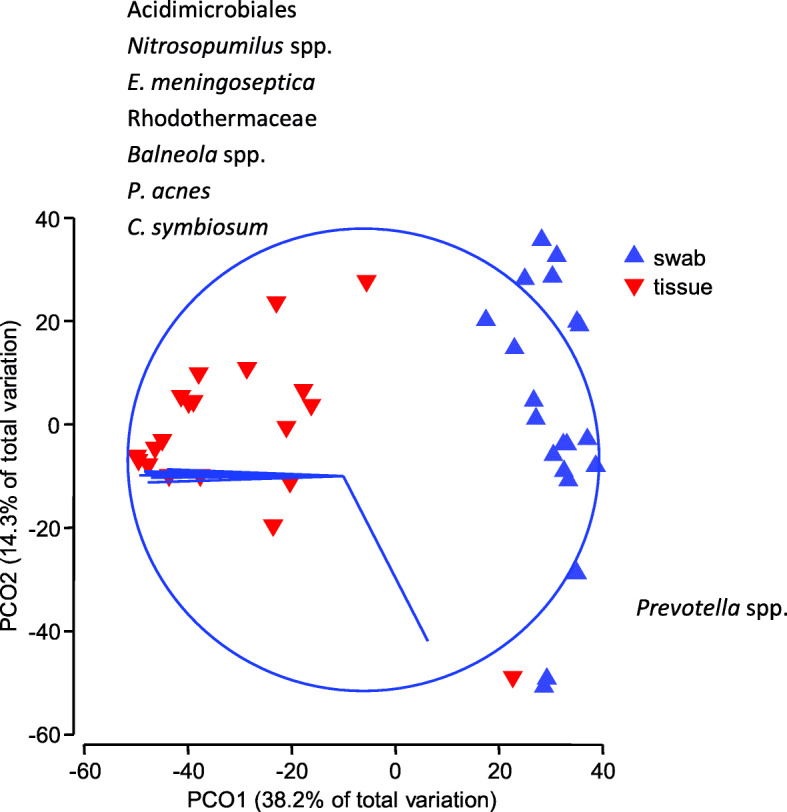
Principle co-ordinate ordination of the bacterial communities from paired DFU swab and tissue biopsies. A Bray Curtis similarity matrix was generated from 4th root-transformed OTUs. Vector overlay is based on Pearson correlation (r ≥ 0.8) with the specific bacteria listed that correspond to the vectors in each direction. Relative abundances of bacterial communities contributing to the disparity along PCO2 were equal to or less than 1% of total reads (Supplementary Table S4 Additional File 2)

Multivariate permutational analysis of variance (PERMANOVA) on the 4th root transformed reads confirmed there was a significant difference in the overall bacterial communities that were sequenced from swabs compared to tissue biopsies (Pseudo F = 18.58, *p* = 0.0001 from 9918 permutations). The principal co-ordinate ordination (PCO) plot reveals clear separation of the swab and tissue samples along PCO1 and substantial variability between the communities of bacteria found in DFUs within each sampling method along PCO2 (Fig. 4). Vector overlay based on Pearson correlation (r ≥ 0.8) indicates that tissue samples are associated with increased numbers of relatively uncommon organisms including the Archaea family Cenarchaeaceae, and bacteria including the species *Elizabethkingia meningosepticum*, the order Acidimicrobiales, *Balneola* spp. and Rhodothermaceae families (Fig. 4). Swabs are characterised by relatively high loads of *Prevotella* spp. (Fig. 4). Swabs and tissue are clustered in separate distinct communities indicating no concordance between the collection methods. RELATE analyses based on matched resemblance matrices for swabs and tissue biopsy samples confirmed that there was no significant multivariate correlation between bacterial communities in paired samples (sample statistic Rho = 0.043, *p* = 0.34 and 9999 permutations of which 3395 were greater than or equal to Rho). The dendogram indicated no pairing of swabs and tissue bacterial communities from the same ulcer (Supplementary Table S5 Additional File 3).

## Discussion

Using 16S rRNA gene sequencing, swabs of DFUs collected using the Levine method were found to effectively detect bacteria in genera with known pathogens, at a similar or higher frequency to those recovered from tissue biopsy samples. Both collection techniques identified the same range of pathogenic genera, although significant differences in relative abundances were detected for some genera and low concordance was displayed between paired swab and biopsy samples overall. Swabs recovered higher relative abundances of common bacteria from genera with known pathogens, whereas uncommon organisms (0.05 to 1% of relative abundance) were more prevalent in tissue biopsies. Therefore, from a clinical perspective, the results of this study suggest that non-invasive swabs are suitable for detecting potential pathogens in DFUs, using 16S rRNA gene sequencing techniques, whereas from a research perspective the higher species richness and diversity in tissues may be of interest.

A higher proportion of wound swab samples detected clinically relevant bacteria from genera of known pathogens, suggesting that bacterial profiling using 16S rRNA gene sequencing from wound swabs could be more reliable for pathogen detection than sequencing tissue biopsies of the wounds. Although other studies have reported a higher number of pathogens isolated from tissue biopsies versus swabs using traditional culture [18–20], the multicentre study by Nelson et al., (2016) also included a small sub study comparing 12 paired swab and tissue samples using PCR [19]. These authors found that in contrast to the results from culture techniques, more pathogens were identified from swabs than tissue samples, when both were analysed by PCR [19]. This lends support to our study conclusions using DNA sequencing. However, these results are in contrast with a study by Dunyach-Remy et al., (2014) using polymerase chain reaction denaturing gradient gel electrophoresis (PCR-DGGE) [24], whereby significantly greater proportions of known pathogens were identified in DFU tissue samples (87%) compared to swab samples (58%), with no pathogenic bacteria detected in 9 of the 20 swab samples. Polymicrobial infections were identified in 16 of the 20 tissue biopsy samples. PCR-DGGE does not provide the same depth of information for pathogen identification as next generation sequencing. Forty seven species were identified from 20 paired DFU samples in the PCR-DGGE study [24], compared to a richness of 185 OTUs represented over 20 paired samples from the present study using 16S rRNA gene sequencing. Furthermore, *P. aeruginosa, Staphylococcus* spp. (other than *S. aureus*), *Corynebacterium* spp. and some members of the Enterobacteriaceae family were not included as pathogens in the previous DGGE study, but were described as intermediate or commensal flora [24]. A study by Frank, Wysocki [25] compared fifteen paired swabs and tissue biopsies from mixed wound types and aetiologies using 16S rRNA gene sequencing and employing an ARB database [26]. Similar to our study, these researchers detected *Streptococcus* spp. more frequently in swabs, and also found that the percentage of occurrence was higher in swabs for *Enterococcus* spp. [25]. However, in contrast to our results, Frank et al. (2009) detected a higher occurrence of *S. aureus*, *Corynebacterium striatum* and *Anaerococcus lactolyticus* in tissue samples compared to swabs. These previous studies comparing wound swabs and tissues used different DNA extraction and bacterial profiling procedures to those used in the current study, with two papers not describing the swab collection method. Our study demonstrates that by using the Levine method with 16S rRNA gene bacterial profiling, swabs can capture a large microbiome from DFUs and in particular, provide good recovery of clinically relevant pathogens when compared to tissue biopsies.

Despite recovering similar pathogens, there was very low concordance in the bacterial profiles between paired swabs and tissue biopsies based on correlation data. The multivariate principle coordinate ordination (PCO) showed a clear segregation of swabs and tissue samples, with no pairing according to the wound of origin. This indicates that the two collection techniques produced different sample populations of microbes. It is possible that the observed differences in the swab and tissue biopsy sample communities could have resulted from the different kits used to extract DNA from the swab and tissue biopsy samples. Further studies should be undertaken using an orthogonal design with standardised extraction methods optimised for both sample types. In this study we standardised the general procedure, however, the lytic mixtures used were optimised for the type of sample. Procedural artefacts could be created by using the same reagents if not suited to the sample. For example a harsher lytic mix may extract bacterial DNA well from tissue but destroy more bacterial DNA in swabs. Conversely a milder lytic mix may not penetrate the tissue sample well and result in under-representation of wound pathogens.

Uncommon organisms identified only in the tissue samples in abundances of up to 1% of total tissue reads included Archaea, which have been previously found in the human gut [27, 28]; cyanobacteria (blue-green algae including *C. symbiosum* identified at a similarity of 100%) usually found in water and produce metabolites potentially toxic to humans and animals [29]; *Chloroflexi* (green non-sulphur bacteria) previously reported as an open sea water residing bacteria that can degrade terrestrial organic matter [30]; *Deinococci*, which are radiation tolerant bacteria and have been described as extremophilic, as the organisms are very resistant to environmental hazards [31]; the hyperthermophilic bacteria Thermotogaceae family which are also extremophiles, preferring temperatures in excess of 45 °C [32]; the Balneolaceae family in the Bacteroidetes phylum [33] and the Rhodothermaceae family also within the Bacteroidetes phylum, which are known as environmental bacteria. The order Acidomicrobiales in the Actinobacteria phylum are found in mixed environments including soil, marine and land water [34]. None of these bacteria were detected in tissue positive controls with reads > 1 (Supplementary Table S7 Additional File 5), and they have not been previously documented in association with human wound infections. It remains possible that some of the distinct OTUs with low relative abundance are contaminants as they have been previously reported from DNA extraction kits [35]. Alternatively they may play an as yet unidentified role in the establishment or maintenance of complex biofilm communities. Therefore, the identification of these environmental organisms in tissue biopsies cannot be disregarded as the microbiome of DFUs is complex, and subsequent studies may confirm or otherwise their significance in maintaining chronicity in these wounds. Further studies aimed at characterising these organisms could be undertaken using tissue samples to ensure maximum representation in the microbiome.

*Elizabethkingia meningoseptica* was identified only from tissue samples at a similarity score of 100%. This bacterium has been previously associated with severe infections, especially in immunocompromised individuals, although the pathogenicity of *E. meningoseptica* has not been described in diabetic wounds [36]. *Cutibacterium acnes* formerly *Propionibacterium acnes* [37] resides in sebaceous follicles on the human skin and was only recovered from DFU tissue biopsies (at a similarity score of 100%) but not swab samples. This organism has been previously detected in DFUs [38, 39]. Obligate anaerobes *Prevotella* spp., *Peptoniphilus* spp., *Peptostreptococcus* spp., *Porphyromonas* spp. and *Bacteroides* spp. were recovered from both swabs and tissue samples and these organisms are known to be involved in human infections, including wounds [38, 40–42].

There was significantly more eukaryotic DNA present in swab samples relative to tissue biopsy samples (Table 1) with many swabs heavily bloodstained after collection from participants. Glassing et al., (2015) discussed competitive inhibition due to large amounts of human DNA on low bacterial biomass samples which can interfere with applications including PCR and 16S rRNA sequencing [43]. A higher ratio of bacterial to human DNA in the tissue biopsy samples in our study may have contributed to the increased richness and diversity in tissue biopsy samples relative to swab samples. In future sampling, care should be taken to reduce the amount of blood on swab samples.

Macro-scale spatial variation, whereby organisms within the wound are non-uniformly distributed, may lead to sampling limitations and therefore differences in community profiles [44–46]. During sample collection in this study sampling positions of the swab and tissue biopsy within each wound differed as the tissue was collected from an unswabbed area. Wound biopsies were collected from a single defined point at the wound edge. By comparison, swabs were rotated to cover more wound surface area. More specifically, using the Levine method, swabs are rotated within a 1 cm^2^ area and to a depth of at least 3 mm depending on the softness of the wound tissue to enhance recovery of the exudate fluid [47]. The greater sample area could be one reason why we found a higher number of reads and pathogens in the swabs compared to biopsies. Nevertheless, while biopsies may only sample one relatively small area, they recover microbes that penetrate deeper into tissue. In a study using confocal laser scanning microscopy (CLSM) on tissue biopsies, *P. aeruginosa* was detected deeper into the tissue relative to *S. aureus* [45]. The association with the surface of wound beds may contribute to why our swab samples had a significantly higher mean relative abundance of *Staphylococcus* spp. than tissue samples, whereas *Pseudomonas* spp. displayed a non-significant trend towards a higher mean relative abundance in tissue biopsies relative to swabs and were found in nine of 20 samples in tissue but in only six swab samples. Composite samples, whether swabs or tissue biopsies, from different sites within a wound, may provide a more consistent and reliable representation of the overall wound microbiome [44].

Despite an apparent lack of concordance in the specific communities detected in paired swabs and tissue biopsies, both methods were able to recover a comparable and diverse range of DFU pathogens. A similar composition of pathogenic and potentially pathogenic organisms in DFUs were reported from a retrospective, multicentre study which used the 16S rRNA gene to sequence and investigate the microbiome of 910 debrided tissue samples from DFUs [12]. Of the seven most abundant bacterial genera (which accounted for approximately 60% of the taxa reported from the retrospective study), all were identified in both swab and tissue biopsy samples from our study. The remaining genera identified and described in the multi-centre study [12], were also identified from a number of tissue biopsy and swab samples used in this study which indicates that our 20 swab and tissue biopsy samples taken from a high risk foot clinic in Sydney, Australia are generally representative of what has been detected in tissue biopsied from a large number of DFUs in other worldwide locations. This information provides confidence in the use of swab samples for future studies aiming to identify the microbiome in DFUs.

## Conclusions

Using 16S rRNA gene sequencing, this study has investigated the concordance of paired swab and tissue biopsy samples from diabetic foot ulcers. The results of univariate and multivariate analyses of identified microbes indicate low concordance using the two collection techniques, which could be the result of within wound macro-scale spatial variation or different lytic mixtures used to extract bacterial DNA. Significantly higher relative abundances of some pathogens were identified in swabs compared to tissue biopsies, although biopsies produced richer microbiomes. From a practical and ethical viewpoint, wound swabs are preferable to tissue biopsies. The high representation of genera containing potential pathogens identified from non-invasive swabs suggests that researchers profiling wound microbiomes could readily collect swab samples using the Levine method, with confidence that the detection of common pathogenic genera will be similar to those obtained from tissue biopsies. Should standard microbiology laboratories adopt molecular based techniques, then wound swabs may prove highly beneficial for clinical practice, minimising the requirement to obtain an invasive tissue biopsy. Swabs collected using the Levine method can easily be used to provide intra- wound and serial wound samples with minimal or no discomfort to the patient and also facilitate between study comparisons using a standardised sample collection technique. From a research perspective further understanding of the microbiome of the DFU is crucial, to facilitate identification of specific organisms which could be responsible for prolonging the healing process. This study indicates that the microbial diversity and community structure obtained from tissue samples may be more representative of the microbiome from the deeper wound bed in DFUs.

## Methods

### Participants and sample collection

To investigate the microbiomes captured by swabs and tissue biopsies 20 patients presenting with an active DFU were recruited from a tertiary referral hospital (Liverpool Hospital High Risk Foot Service Sydney Australia) over a six-month study period. At the time of presentation, DFUs were either clinically infected as per the Infectious Disease of America guidelines for diabetic foot infection (DFI) [2] or chronic, non-healing and not responding to routine care including offloading, revascularisation and compression therapy. All participants had been diagnosed with peripheral neuropathy. One participant had been diagnosed with ischaemic heart disease (IHD). Participants receiving systemic or topical antimicrobials two weeks prior to this study were not included. Demographic data is provided in Supplementary Table S6 Additional File 4.

Ethics approval for the study was granted by the South West Sydney Local Health District Research and Ethics Committee (HREC/14/LPOOL/487, SSA/14/LPOOL/489) and all participants provided informed written consent.

### Collection of tissue biopsies

Tissue biopsies 3 mm wide and 10 mm deep were collected using a biopsy punch from the edge of each DFU after cleansing the wound with 0.9% NaCl. After aseptic removal, tissue biopsy samples were washed in a PBS bath to remove coagulated blood and surface bacteria. Samples were sectioned transversely into a 1.5 mm fragment and placed into approximately 100 μl of RNAlater (Sigma-Aldrich Australia cat. no. R 0901) for 24 h at 4 °C and frozen at − 80 °C until DNA extraction.

### Collection of swab samples using Levine method

Swabs (Copan- Amies without charcoal M40 transystem Interpath Services, Heidelberg West Victoria, Australia) were collected from all DFUs using the Levine method, which involves rotating the swab over a normal saline cleansed area of 1 cm^2^ for 5 s and applying enough pressure to exude and collect fluid from the tissue onto the swab [17, 47]. Swab samples were collected from an area close to the edge of the ulcer from where tissue biopsy samples were collected from study participants. All swab samples were frozen at − 20 °C during transport to and storage at Southern Cross University Lismore N.S.W. before DNA extraction.

### Extraction of DNA

Slightly different procedures were used for extraction of bacterial DNA from tissue biopsies and swab samples in order to maximise the recovery of pathogens according to the nature of the sample. For both types of samples, bead-beating was used prior to extraction to maximise the ability to extract DNA out of the cellular material. However, the lytic mixtures used were optimised for the type of sample based on established protocols.

### Extraction of DNA from tissue biopsies

Approximately 5–10 mg of DFU tissue was defrosted on ice prior to genomic DNA extraction using MoBio Power Biofilm isolation kit (MoBio, Carlsbad, MA, United States) as per manufacturer’s instructions. RNAlater was removed using a sterile pipette. Briefly, the tissue sample was added to a PowerBiofilm bead tube containing 350 μl lysis solution and 100 μl of a chaotropic solution and heated at 65 °C for 5 min. Bead tubes were homogenised at 3200 rpm (940 g) or 53 Hz for 30 s and centrifuged for 1 min at 11900 rpm (13,000 g) enabling dissolution of the biofilm matrix and microbial cell lysis using chemical and physical conditions. The supernatant was transferred to a 2 ml collection tube, to which was added a high salt solution and the mixture poured through silica based spin filter and centrifuged. The flow through was discarded. This step was repeated and the DNA was eluted using sterile 10 mM Tris into a clean 2 ml microtube. The extracted tissue biopsy DNA samples were frozen and stored at − 20 °C.

### Extraction of DNA from swabs

The following method was modified from a previously described protocol [44]. Frozen swabs were thawed to room temperature before extraction. A lytic mix was prepared daily before extractions using the following concentrations of enzymes: 25 μl of 20 mg/ml Lysozyme (chicken egg white Sigma-Aldrich Australia cat.no. L6876), 25 μl of 100 U/ml Mutanolysin (*Streptomyces globisporus* ATCC 21553 Sigma-Aldrich Australia cat.no. M9901), 20 μl of 40 μg/ml Lysostaphin (*Staphylococcus staphylolyticus* Sigma Aldrich Australia cat.no. L7386) and 930 μl DNase and RNase free water (Sigma-Aldrich, Australia) to make a final volume of 1000 μl.

Thawed swab buds were each placed in sterile labelled 1.5 ml micro tubes containing 300 μl of DNase and RNase free water (Sigma), 50 μl of lytic mix and incubated at 37 °C for 30 min in a dry heating block. Each swab was removed and the sample lysate centrifuged for 1 min at 1500 rpm (206 g). The sample lysate was aseptically transferred to a 2 ml bead beating tube containing 6 × 2.8 mm ceramic beads (Mobio, Carlsbad, MA, United States cat. no.13114–325). Bead beating at 25 Hz (Qiagen Tissue Lyser 11, Hilden, Germany) for 1 min was performed, after which the bead tubes were centrifuged at 1200 rpm (132 g) for 1 min. The sample lysate from the bead tube was aseptically transferred to a sterile 1.5 ml tube containing 200 μl Qiagen Buffer AL (QIAamp extraction kit for blood and body fluids, Hilden, Germany cat. no.51304) and vortexed for 10 s, after which 20 μl of 20 mg/ml Proteinase K (QIAamp extraction kit for blood and body fluids) was added and the sample lysate vortexed for another 15 s. The sample lysate was incubated for 10 min at 56 °C in a dry heating block and centrifuged for 1 min at 1500 rpm (206 g). Following these steps, the procedure described in the QIAamp DNA purification from Blood and Body fluids (spin protocol) instructions were followed from step 6 to step 10 and briefly involved progressive ethanol washing (two steps) and centrifugation using spin columns with silica membranes designed to capture the DNA while proteins and other contaminants were discarded with the ethanol in the filtrate. A further two steps involved washing the DNA in buffer and discarding the buffer filtrate. Extracted DNA samples were then incubated in 200 μl AE buffer at room temperature for 5 min before centrifuging at 8000 rpm (5867 g) for 1 min, as per manufacturers’ instructions to increase DNA yields. The extracted swab DNA samples were frozen and stored at − 20 °C.

The absorbance of the extracted tissue biopsy and swab DNA at 260 nm and 280 nm was measured using the Nanodrop 2000 (Thermo Fisher Scientific, Waltham, Massachusetts, United States) and samples with a 260/280 ratio of ≥1.3 were used for analysis.

Although different extraction kits were used for samples investigated in this study, the DNA extraction procedures for the tissue biopsy and swab samples were based on similar principles. These procedures included chemical and mechanical (bead beating) lysis of the bacterial cells, removal of cell debris including proteins and polysaccharides, and use of a silica spin column to recover sample bacterial DNA which was subsequently washed and eluted. However the lytic mixtures differed between the two extraction methods and the results are interpreted with this in mind.

### Quantitative PCR (qPCR) to determine bacterial abundance and 16S/18S ratios

qPCR was carried out using a 25 μl reaction mixture containing 1X Brilliant II Sybr Green qPCR Master mix (Stratagene, San Diego, CA, Bacterial primers targeting 16S rRNA gene 341F (5′- CCTACGGGAGGCAGCAG − 3′) and 534R (5′- ATTACCGCGGCTGCTGG − 3′), to which 2 μl of extracted DNA template was added. The cycling conditions were as follows: 95 °C for 10 min, followed by 40 cycles of 95 °C for 15 s, 56 °C for 30 s and 72 °C for 30 s, using Stratagene MX 3000P (Agilent Technologies, CA, United States). Each qPCR was run with genomic standards comprising 10^3^–10^6^ copies/μl DNA extracted *Staphylococcus aureus* ATCC 25923. Microbial DNA free water (2 μl) (Qiagen Hilden Germany cat.no.338132) was used as the negative template control (NTC). The number of 16S rRNA gene copies/μl for each sample was determined and used to calculate abundance of bacteria in each sample. Genomic standards of 10 ^3^–10^6^ copies/μl of DNA extracted from fresh human tissue were prepared to determine the number of copies of the eukaryotic 18S rRNA gene, to ascertain the ratio of human tissue in biopsies relative to that found in swabs.

### Bacterial community profiling

Extracted DNA from tissue biopsies and wound swabs was sent to a commercial laboratory for analysis (Australian Centre for Ecogenomics, Brisbane, Australia), where identical primers for both swabs and tissue were employed for community profiling in the V4 region (515F 5′- GTGYCAGCMGCCGCGGTAA − 3′ and 806wR 5′- GACTACHVGGGTWTCTAATCC-3′) [48] modified to contain Illumina specific adaptor sequence: (515F: 5′- TCGTCGGCAGCGTCAGATGTGTATAAGAGACAGGTGYCAGCMGCCGCGGTAA − 3′ and 803Rb: 5′- GTCTCGTGGGCTCGGAGATGTGTATAAGAGACAGGACTACHVGGGTATCTAATCC − 3′) The prokaryotic primer pair Prok SSU 515F 806Rb amplifies the small subunit (SSU) ribosomal RNA of bacteria and Archaea (16S), specifically the V4 region. In *Escherichia coli*, it amplifies the 515–806 region of the 16S rRNA gene.

Preparation of the 16S library was performed as described using the workflow outlined by Illumina (2013). The PCR products of ~ 466 bp were first amplified according to the workflow specified, but with substitution of the polymerase to NEBNext® Ultra™ II Q5® Mastermix (New England Biolabs #M0544). Agencourt AMPure XP beads (Beckman Coulter) were used to purify the PCR amplicons, which were then indexed with unique 8 bp barcodes using the Illumina Nextera XT 384 sample Index Kit A-D (Illumina FC-131-1002). Indexed amplicons were pooled in equimolar concentrations and sequenced using paired end sequencing with V3 300bp chemistry on a MiSeq Sequencing System (Illumina) in the Australian Centre for Ecogenomics according to manufacturer’s protocol.

Control reactions used for the amplicon library construction and sequencing included:

- Positive control amplification of a mock community to test for bias in the amplicon library construction.
- Negative amplicon control from a processed reagent to test for potential contamination during library construction.
- Empty chamber controls from single wells within processing plates to test for potential cross contamination within the library preparation.
- In line controls using negative index positions between runs to test for any bleed through from run to run.

Prior to data processing, resulting sequences with over 10,000 raw reads per sample were determined to pass Quality Control (QC) and overall > 70% passed QC metrics of Q30 for 600 bp reads, in line with Illumina supplied reagent metrics.

### Sequence analysis

Raw sequencing data in FASTQ format was checked for quality using the FastQC tool [49]. Trimmomatic software (version 0.38) [50] was used to remove primer and low quality sequences. Both paired end reads and forward reads were generated from the Illumina MiSeq. After quality control only the forward reads with > 200 bp lengths were retained for further analysis. Sequence analysis was performed by means of the ACE mitag pipeline which used QIIME (version 1.8) [51] and pick_open_reference_OTUs.py workflow. UCLUST [52] clustering algorithm was used to cluster the reads into operational taxonomic units (OTUs). Singletons and OTUs with less than < 0.05% abundance were filtered out. OTUs were BLAST searched against the Greengenes chimera checked 16S rRNA gene reference database [53] (version 2013/05) for 16S using ≥97% similarity score.

### Statistical analyses

The DIVERSE function in PRIMER V7 version 7.0.13 + PERMANOVA (PRIMER-E Ltd., Plymouth, UK) was used to generate species richness (S), and Shannon’s diversity index (H) based on the reads data for each sample. Rarefaction of reads to 1500 was accepted to provide a consistent depth of coverage. Samples with < 1500 reads were excluded from further analysis. Univariate paired sample analyses were then undertaken using GraphPad Prism version 7.02. The number of copies of the 16S rRNA gene/μl was used to determine the bacterial abundance in each sample. The number of reads and bacterial abundance (qPCR) data did not pass D’Agostino and Pearson normality tests, therefore, non-parametric analyses using Wilcoxon signed-rank tests were performed. Species richness data (number of distinct OTUs) and Shannon’s H index data passed normality testing and were analysed using parametric paired sample t-tests. Spearman rank correlations were performed between paired samples to establish whether there was concordance in the order in which swab and tissue samples were ranked for the number of reads, species richness and Shannon’s H index. The dataset was filtered to select genera with known pathogens -namely *Staphylococcus* spp., *Streptococcus* spp., *Enterococcus* spp., *Pseudomonas* spp. and *Corynebacterium* spp., the Enterobacteriaceae family, and members of the Clostridiales family XI (*Finegoldia* spp. and *Anaerococcus* spp.) [10]. The mean relative abundance of reads in each of these groups was square root transformed (sqrt) to reduce heterogeneity of variance and analysed using the Wilcoxin signed rank test. Spearman rank correlation was used for all groups. Results were considered significant for *p* < 0.05. Comparison of the bacterial community composition in swabs versus tissue samples was undertaken using PRIMER V7 version 7.0.13 + PERMANOVA. A Bray Curtis similarity matrix was constructed from 4th root transformed data (to down-weight overly abundant taxa), with a Dummy variable of 1. A one factor permutational analysis of variance (PERMANOVA) was run to compare swabs and tissue samples, with 9999 permutations. Principle coordinate ordination plots were used to graphically display the difference between swabs and tissue samples, with vector overlay based on Pearson correlation greater than 0.8. Dendograms were produced from hierarchical cluster analysis to investigate the degree of pairing in bacterial communities of samples taken from the same DFU. To test for concordance in the communities from the same DFU, separate similarity matrices were prepared on the standardised swab and tissue samples. The RELATE function was used to test the matched resemblance matrices for paired samples based on Spearman Rank correlation.

### Positive and negative controls for tissue biopsies

Positive controls for the tissue biopsy method included cultures of *S. aureus* and *Acinetobacter rhizosphaerae* prepared to an O.D. of 0.03 at 600 λ absorbance. Dilutions of *S. aureus* and of *A. rhizosphaerae* in 900 μl nuclease free water (Qiagen CAT No. / ID: 129114) were prepared and extracted using the tissue extraction method described. Sequencing results are presented in Supplementary Table S7 Additional File 5 and were used to confirm or otherwise that reagent contamination was not biasing results.

Negative control tissue biopsy method: Sequencing results indicated no contamination of the positive controls (Supplementary Table S7 Additional File 5) and hence of the extraction procedure. This result acted as a negative control for the DNA extraction from tissue biopsies.

### Positive and negative controls for swabs

Positive controls: Twenty four hour colonies of *S. aureus* ATCC 29213 and *Pseudomonas aeruginosa* ATCC 27856 were prepared in nutrient broth (Biomerieux, Australia) and diluted in DNase and RNase free water (Sigma-Aldrich, Australia) to an O.D. of 0.03 using 600 λ absorbance. A 1:5 dilution of each control organism was prepared using 250 μl DNase and RNase free water (Sigma-Aldrich, Australia) and 50 μl of each control organism and extracted using the described DNA swab extraction method. Sequencing results are presented in Supplementary Table S8 Additional File 5 and were used to confirm or otherwise that reagent contamination was not biasing results.

Negative controls to detect any sample collection swab contamination were prepared with an unused thawed frozen Copan swab and extracted using the described swab procedure. Sequencing results are presented in Supplementary Table S9 and Supplementary Table S10 Additional File 5 and were used to confirm or otherwise that swab contamination was not biasing results.

Negative controls to detect reagent contamination with no added sample were performed for the swab DNA extraction method with reagents only and followed the relevant DNA extraction procedures with 300 μl of DNase and RNase free water (Sigma-Aldrich, Australia) and 50 μl of lytic mix placed in a sterile 1.5 ml microtube and the swab DNA extraction procedure followed exactly. Sequencing and summarised results are presented in Supplementary Table S9 and Supplementary Table S10 Additional File 5 and were used to confirm or otherwise that swab and/or reagent contamination was not biasing results. Quantitative PCR results for swab controls are presented in Supplementary Table S11 Additional File 5.

## Supplementary information

**Additional file 1 Supplementary Table S1.** Number of reads and copies of 16S rRNA gene/μl in swab and tissue biopsy samples. **Supplementary Table S2.** Spearman Rank correlations between swab and tissue biopsy samples. **Supplementary Table S3.** No. of distinct OTUs (Richness) pre and post rarefaction at 1500 reads.

**Additional file 2 Supplementary Table S4.** Summary of organisms, total reads and relative abundances in swabs and tissue biopsy samples

**Additional file 3 Supplementary Table S5.** Dendogram from hierarchical cluster analysis of bacterial communities indicating no clustering

**Additional file 4 Supplementary Table S6.** Demographics of 20 participants

**Additional file 5 Supplementary Table S7.** Positive control data (tissue biopsies) using tissue biopsy DNA extraction method. **Supplementary Table S8.** Positive control data (swabs). **Supplementary Table S9.** Negative control data (swabs). **Supplementary Table S10**. Copan swab control data. **Supplementary Table S11.** Quantitative PCR data for positive and negative swab controls

## Data Availability

Data generated or analysed during this study are all included in this published article and its additional information files.

## Abbreviations

DFUs: Diabetic foot ulcers
PCR: Polymerase chain reaction
qPCR: Quantitative polymerase chain reaction
OTUs: Operational taxonomic units

## References

[1] Futrega K, King M, Lott WB, Doran MR (2014). Treating the whole not the hole: necessary coupling of technologies for diabetic foot ulcer treatment. Trends Mol Med.

[2] Lipsky BA, Berendt AR, Cornia PB, Pile JC, Peters EJ, Armstrong DG (2012). Infectious Diseases Society of America: clinical practice guideline for the diagnosis and treatment of diabetic foot infections 2012. Clin Infect Dis.

[3] Watters C, Yuan TT, Rumbaugh KP. Beneficial and deleterious bacterial-host interactions in chronic wound pathophysiology: University of Texas at Austin Austin United States; 2015.

[4] Gardner SE, Hillis SL, Heilmann K, Segre JA, Grice EA (2013). The neuropathic diabetic foot ulcer microbiome is associated with clinical factors. Diabetes..

[5] Lavigne J-P, Sotto A, Dunyach-Remy C, Lipsky BA. New molecular techniques to study the skin microbiota of diabetic foot ulcers. Adv Wound Care. 2015;4(1):38–49. 10.1089/wound.2014.0532 PMC4281861 25566413

[6] Tuttle MS, Mostow E, Mukherjee P, Hu FZ, Melton-Kreft R, Ehrlich GD, et al. Characterization of bacterial communities in venous insufficiency wounds using conventional culture and molecular diagnostic methods. Journal of Clinical Microbiology. 2011:JCM. 00847–00811. 10.1128/JCM.00847-11 PMC3209111 21880958

[7] Gardner SE, Frantz RA, Doebbeling BN (2001). The validity of the clinical signs and symptoms used to identify localized chronic wound infection. Wound Repair Regen.

[8] Esposito S, Noviello S, De Caro F, Boccia G (2018). New insights into classification, epidemiology and microbiology of SSTIs, including diabetic foot infections. Infez Med.

[9] Percival SL, Malone M, Mayer D, Salisbury AM, Schultz G. Role of anaerobes in polymicrobial communities and biofilms complicating diabetic foot ulcers. Int Wound J. 2018;15(5):776–82. 10.1111/iwj.12926 PMC7950146 29863794

[10] Malone M, Johani K, Jensen S, Gosbell I, Dickson H, Hu H (2017). Next generation DNA sequencing of tissues from infected diabetic foot ulcers. EBioMedicine..

[11] Loesche M, Gardner SE, Kalan L, Horwinski J, Zheng Q, Hodkinson BP (2017). Temporal stability in chronic wound microbiota is associated with poor healing. J Invest Dermatol.

[12] Wolcott RD, Hanson JD, Rees EJ, Koenig LD, Phillips CD, Wolcott RA, et al. Analysis of the chronic wound microbiota of 2,963 patients by 16S rDNA pyrosequencing. Wound Repair Regen. 2016;24(1):163–74. 10.1111/wrr.12370 26463872

[13] Smith K, Collier A, Townsend EM, O’Donnell LE, Bal AM, Butcher J (2016). One step closer to understanding the role of bacteria in diabetic foot ulcers: Characterising the microbiome of ulcers. BMC Microbiol.

[14] Sandhu S, Rathnayake IU, Huygens F (2014). Prevalence of methicillin resistance and virulence determinants of *Staphylococcus aureus* in diabetic foot ulcers. Int J Basic Clin Pharmacol.

[15] Huang Y, Cao Y, Zou M, Luo X, Jiang Y, Xue Y, et al. A comparison of tissue versus swab culturing of infected diabetic foot wounds. Int J Endocrinol. 2016;2016. 10.1155/2016/8198714 PMC4829715 27123004

[16] Mutluoglu M, Uzun G, Turhan V, Gorenek L, Ay H, Lipsky BA (2012). How reliable are cultures of specimens from superficial swabs compared with those of deep tissue in patients with diabetic foot ulcers?. J Diabetes Complicat.

[17] Levine NS, Lindberg RB, Mason AD, Pruitt BA (1976). The quantitative swab culture and smear: a quick, simple method for determining the number of viable aerobic bacteria on open wounds. J Trauma Acute Care Surg.

[18] Backhouse M, Nelson A, Wright-Hughes A, Bhogal M, Brown S, Reynolds C (2015). Concordance in diabetic foot infection: agreement in reported presence of likely pathogens in swabs and tissue samples from infected diabetic foot ulcers. J Foot Ankle Res.

[19] Nelson EA, Wright-Hughes A, Brown S, Lipsky BA, Backhouse M, Bhogal MS, et al. Concordance in diabetic foot ulceration: a cross-sectional study of agreement between wound swabbing and tissue sampling in infected ulcers. Health Technol Assess. 2016:1–176. 10.3310/hta20820 PMC5116580 27827300

[20] Nelson A, Wright-Hughes A, Backhouse MR, Lipsky BA, Nixon J, Bhogal MS (2018). CODIFI (concordance in diabetic foot ulcer infection): a cross-sectional study of wound swab versus tissue sampling in infected diabetic foot ulcers in England. BMJ Open.

[21] Lipsky BA, Aragón-Sánchez J, Diggle M, Embil J, Kono S, Lavery L (2016). IWGDF guidance on the diagnosis and management of foot infections in persons with diabetes. Diabetes Metab Res Rev.

[22] Høiby N, Bjarnsholt T, Moser C, Bassi G, Coenye T, Donelli G (2015). ESCMID guideline for the diagnosis and treatment of biofilm infections 2014. Clin Microbiol Infect.

[23] Grice EA, Kong HH, Renaud G, Young AC, Bouffard GG, Blakesley RW (2008). A diversity profile of the human skin microbiota. Genome Res.

[24] Dunyach-Remy C, Cadière A, Richard J-L, Schuldiner S, Bayle S, Roig B (2014). Polymerase chain reaction–denaturing gradient gel electrophoresis (PCR–DGGE): a promising tool to diagnose bacterial infections in diabetic foot ulcers. Diabetes Metab.

[25] Frank DN, Wysocki A, Specht-Glick DD, Rooney A, Feldman RA, St Amand AL (2009). Microbial diversity in chronic open wounds. Wound Repair Regen.

[26] Ludwig W, Strunk O, Westram R, Richter L, Meier H, Yadhukumar, et al. ARB: a software environment for sequence data. Nucleic Acids Res 2004;32(4):1363–1371. 10.1093/nar/gkh293 PMC390282 14985472

[27] Nkamga VD, Henrissat B, Drancourt M. Archaea: Essential inhabitants of the human digestive microbiota. Hum Microbiome J. 2017;3:1–8.

[28] Lurie-Weinberger MN, Gophna U (2015). Archaea in and on the human body: health implications and future directions. PLoS Pathog.

[29] Otten TG, Paerl HW (2015). Health effects of toxic cyanobacteria in US drinking and recreational waters: our current understanding and proposed direction. Curr Environ Health Rep.

[30] Colatriano D, Tran P, Gueguen C, Williams W, Lovejoy C, Walsh D. Genomic evidence for the degradation of terrestrial organic matter by pelagic Arctic Ocean *Chloroflexi* bacteria. BioRxiv. 2018;325027. 10.1038/s42003-018-0086-7 PMC6123686 30271971

[31] LaGier MJ. Predicted cold shock proteins from the extremophilic bacterium *Deinococcus maricopensis* and related *Deinococcus* species. Int J Microbiol. 2017;2017:1–10. article ID 5231424. 10.1155/2017/5231424. 10.1155/2017/5231424 PMC5624153 29098004

[32] Pollo SM, Zhaxybayeva O, Nesbø CL (2015). Insights into thermoadaptation and the evolution of mesophily from the bacterial phylum Thermotogae. Can J Microbiol.

[33] Xia J, Ling S-K, Wang X-Q, Chen G-J, Du Z-J (2016). *Aliifodinibius halophilus* sp. nov., a moderately halophilic member of the genus *Aliifodinibius*, and proposal of Balneolaceae fam. Nov. Int J Syst Evol Microbiol.

[34] Mizuno CM, Rodriguez-Valera F, Ghai R (2015). Genomes of planktonic Acidimicrobiales: widening horizons for marine Actinobacteria by metagenomics. MBio..

[35] Salter SJ, Cox MJ, Turek EM, Calus ST, Cookson WO, Moffatt MF (2014). Reagent and laboratory contamination can critically impact sequence-based microbiome analyses. BMC Biol.

[36] Jean S, Lee W, Chen F, Ou T, Hsueh P (2014). *Elizabethkingia meningoseptica*: an important emerging pathogen causing healthcare-associated infections. J Hosp Infect.

[37] Dréno B, Pécastaings S, Corvec S, Veraldi S, Khammari A, Roques C (2018). Cutibacterium acnes (Propionibacterium acnes) and acne vulgaris: a brief look at the latest updates. J Eur Acad Dermatol Venereol.

[38] van Asten S, La Fontaine J, Peters E, Bhavan K, Kim P, Lavery L (2016). The microbiome of diabetic foot osteomyelitis. Eur J Clin Microbiol Infect Dis.

[39] Rhoads DD, Cox SB, Rees EJ, Sun Y, Wolcott RD (2012). Clinical identification of bacteria in human chronic wound infections: Culturing vs. 16S ribosomal DNA sequencing. BMC Infect Dis.

[40] Buhl M, Willmann M, Liese J, Autenrieth IB, Marschal M (2016). *Prevotella colorans* sp. nov., isolated from a human wound. Int J Syst Evol Microbiol.

[41] Charles PG, Uçkay I, Kressmann B, Emonet S, Lipsky BA (2015). The role of anaerobes in diabetic foot infections. Anaerobe..

[42] Murphy EC, Frick I-M (2013). Gram-positive anaerobic cocci–commensals and opportunistic pathogens. FEMS Microbiol Rev.

[43] Glassing A, Dowd SE, Galandiuk S, Davis B, Jorden JR, Chiodini RJ (2015). Changes in 16s RNA gene microbial community profiling by concentration of prokaryotic DNA. J Microbiol Methods.

[44] Price LB, Liu CM, Frankel YM, Melendez JH, Aziz M, Buchhagen J (2011). Macroscale spatial variation in chronic wound microbiota: a cross-sectional study. Wound Repair Regen.

[45] Fazli M, Bjarnsholt T, Kirketerp-Møller K, Jørgensen B, Andersen AS, Krogfelt KA (2009). Nonrandom distribution of *Pseudomonas aeruginosa* and *Staphylococcus aureus* in chronic wounds. J Clin Microbiol.

[46] Johani K, Malone M, Jensen S, Gosbell I, Dickson H, Hu H, et al. Microscopy visualisation confirms multi-species biofilms are ubiquitous in diabetic foot ulcers. Int Wound J. 2017;14(6):1160–9. 10.1111/iwj.12777 PMC7949972 28643380

[47] Gardner SE, Frantz RA, Saltzman CL, Hillis SL, Park H, Scherubel M (2006). Diagnostic validity of three swab techniques for identifying chronic wound infection. Wound Repair Regen.

[48] Engelbrektson A, Kunin V, Wrighton KC, Zvenigorodsky N, Chen F, Ochman H (2010). Experimental factors affecting PCR-based estimates of microbial species richness and evenness. ISME J.

[49] Bioinformatics B (2011). FastQC A quality control tool for high throughput sequence data.

[50] Bolger AM, Lohse M, Usadel B. Trimmomatic: a flexible trimmer for Illumina sequence data. Bioinformatics. 2014;30(15):2114–20. 10.1093/bioinformatics/btu170 PMC4103590 24695404

[51] Kuczynski J, Stombaugh J, Walters WA, González A, Caporaso JG, Knight R (2012). Using QIIME to analyze 16S rRNA gene sequences from microbial communities. Curr Protocols Microbiol.

[52] Edgar RC (2010). Search and clustering orders of magnitude faster than BLAST. Bioinformatics..

[53] DeSantis TZ, Hugenholtz P, Larsen N, Rojas M, Brodie EL, Keller K (2006). Greengenes, a chimera-checked 16S rRNA gene database and workbench compatible with ARB. Appl Environ Microbiol.

