# Supplementary material for: The microbiome of diabetic foot ulcers: a comparison of swab and tissue biopsy wound sampling techniques using 16S rRNA gene sequencing
Source: BMC Microbiol. 2020 Jun 16;20:163. doi: 10.1186/s12866-020-01843-2 (PMC7296698; doi:10.1186/s12866-020-01843-2)
Supplement: Supplementary file 1 — Additional file 1 Supplementary Table S1. Number of reads and copies of 16S rRNA gene/μl in swab and tissue biopsy samples. Supplementary Table S2. Spearman Rank correlations between swab and tissue biopsy samples. Supplementary Table S3. No. of distinct OTUs (Richness) pre and post rarefaction at 1500 reads. [file 12866_2020_1843_MOESM1_ESM.docx]

**Supplementary Table S1 Additional File 1**

**Number of Reads and number of copies of 16S rRNA gene/µl in swab and tissue biopsy samples N=20**

| Sample no. | #Reads  swabs | Copies of 16S rRNA gene/µl swabs | Sample no. | #Reads  tissue | Copies of 16S rRNA gene/µl  tissue |
| --- | --- | --- | --- | --- | --- |
| swab1 | 7673 | 10900 | tissue1 | 3916 | 255 |
| swab2 | 49717 | 200700 | tissue2 | 1530 | 510 |
| swab3 | 27203 | 2704000 | tissue3 | 16820 | 5348 |
| swab4 | 63099 | 2939000 | tissue4 | 57984 | 5185000 |
| swab5 | 31644 | 23630 | tissue5 | 3718 | 232 |
| swab6 | 28480 | 221000 | tissue6 | 30876 | 745000 |
| swab7 | 29833 | 44750 | tissue7 | 10402 | 105000 |
| swab8 | 20284 | 6626 | tissue8 | 14506 | 3709 |
| swab9 | 26795 | 1524000 | tissue9 | 32347 | 205800 |
| swab10 | 50668 | No result | tissue10 | 4008 | 8604 |
| swab11 | 19322 | 2299000 | tissue11 | 5379 | 16130 |
| swab12 | 32164 | 13090 | tissue12 | 756 | 5375 |
| swab13 | 9760 | 51510 | tissue13 | 28674 | 1786 |
| swab14 | 45499 | 1191000 | tissue14 | 49498 | 24850 |
| swab15 | 40298 | 277800 | tissue15 | 2112 | 198 |
| swab16 | 40754 | 44630 | tissue16 | 586 | 349 |
| swab17 | 38993 | 162500 | tissue17 | 21304 | 353 |
| swab18 | 55889 | 95510 | tissue18 | 9574 | 907 |
| swab19 | 12368 | 14820 | tissue19 | 1827 | 209400 |
| swab20 | 9831 | 21600 | tissue20 | 9293 | 319 |
| **Total no. reads** | **640274** |  |  | **305110** |  |

#Reads in filtered OTU table (>0.05% abundance)

**Supplementary Table S2 Additional File 1**

**Spearman rank correlations between swab and tissue biopsy samples N=20**

|  | Spearman rank  r value | p value |
| --- | --- | --- |
| Abundance (qPCR) | 0.038 | 0.053 |
| No. of reads | -0.012 | 0.960 |
| Bacterial Richness^1^ | 0.118 | 0.619 |
| Shannon's diversity^2^ | -0.130 | 0.585 |
| ^#^Staphylococcus | 0.348 | 0.133 |
| ^#^Enterococcus | 0.257 | 0.275 |
| ^#^Corynebacterium | -0.013 | 0.956 |
| ^#^Enterobacteriaceae | -0.011 | 0.965 |
| ^#^Pseudomonas | 0.01 | 0.966 |
| ^#^Anaerococcus | 0.1 | 0.674 |
| ^#^Finegoldia | 0.207 | 0.381 |

^1^Rarefied data at 1500 reads ^2^Standardised data ^#^ square root transformed values

## Supplementary Table S3 Additional File 1

**No. of distinct OTUs (Richness) pre and post rarefaction at 1500 reads**

| Sample no. | No. of distinct OTUs before rarefaction  swabs | No. of distinct OTUs after rarefaction | Sample no. | No. of distinct OTUs before rarefaction  tissue | No. of distinct OTUs after rarefaction reads |
| --- | --- | --- | --- | --- | --- |
| swab1 | 12 | 11 | tissue1 | 43 | 41 |
| swab2 | 34 | 15 | tissue2 | 39 | 39 |
| swab3 | 50 | 34 | tissue3 | 61 | 39 |
| swab4 | 32 | 22 | tissue4 | 29 | 24 |
| swab5 | 30 | 18 | tissue5 | 46 | 42 |
| swab6 | 40 | 26 | tissue6 | 69 | 37 |
| swab7 | 41 | 29 | tissue7 | 57 | 38 |
| swab8 | 43 | 22 | tissue8 | 68 | 62 |
| swab9 | 38 | 27 | tissue9 | 38 | 16 |
| swab10 | 56 | 39 | tissue10 | 50 | 46 |
| swab11 | 36 | 21 | tissue11 | 56 | 53 |
| swab12 | 43 | 24 | tissue12 | 45 | 45 |
| swab13 | 56 | 41 | tissue13 | 65 | 51 |
| swab14 | 37 | 22 | tissue14 | 43 | 14 |
| swab15 | 46 | 27 | tissue15 | 35 | 34 |
| swab16 | 40 | 19 | tissue16 | 28 | 28 |
| swab17 | 54 | 41 | tissue17 | 54 | 42 |
| swab18 | 36 | 22 | tissue18 | 48 | 43 |
| swab19 | 28 | 19 | tissue19 | 35 | 34 |
| swab20 | 34 | 23 | tissue20 | 47 | 32 |
