# Supplementary material for: The microbiome of diabetic foot ulcers: a comparison of swab and tissue biopsy wound sampling techniques using 16S rRNA gene sequencing
Source: BMC Microbiol. 2020 Jun 16;20:163. doi: 10.1186/s12866-020-01843-2 (PMC7296698; doi:10.1186/s12866-020-01843-2)
Supplement: Supplementary file 2 — Additional file 2 Supplementary Table S4. Summary of organisms, total reads and relative abundances in swabs and tissue biopsy samples [file 12866_2020_1843_MOESM2_ESM.docx]

# **Supplementary Table S4 Additional File 2 Summary of organisms, total reads and relative abundance in swabs and tissue biopsy samples**

| K=kingdom, p=phyla, c=class, o=order, f=family, g=genus, s=species | total Reads | Relative | total reads | Relative |
| --- | --- | --- | --- | --- |
|  | swabs | abundance | tissue | abundance |
| k__Archaea; p__Crenarchaeota; c__Thaumarchaeota; o__Cenarchaeales; f__Cenarchaeaceae; g__; s__ | 0 | N.D. | 195 | <1% |
| k__Archaea; p__Crenarchaeota; c__Thaumarchaeota; o__Cenarchaeales; f__Cenarchaeaceae; g__Cenarchaeum; s__symbiosum | 0 | N.D. | 1847 | <1% |
| k__Archaea; p__Crenarchaeota; c__Thaumarchaeota; o__Cenarchaeales; f__Cenarchaeaceae; g__Nitrosopumilus; s__ | 0 | N.D. | 723 | <1% |
| k__Archaea; p__Euryarchaeota; c__Methanococci; o__Methanococcales; f__Methanococcaceae; g__Methanococcus; s__ | 0 | N.D. | 3617 | 1% |
| k__Bacteria; p__[Thermi]; c__Deinococci; o__Deinococcales; f__Deinococcaceae; g__Deinococcus; s__ | 0 | N.D. | 294 | <1% |
| k__Bacteria; p__[Thermi]; c__Deinococci; o__Thermales; f__Thermaceae; g__Thermus; s__ | 0 | N.D. | 780 | <1% |
| k__Bacteria; p__Acidobacteria; c__Acidobacteria-6; o__BPC015; f__; g__; s__ | 0 | N.D. | 88 | <1% |
| k__Bacteria; p__Actinobacteria; c__Acidimicrobiia; o__Acidimicrobiales; f__; g__; s__ | 0 | N.D. | 358 | <1% |
| k__Bacteria; p__Actinobacteria; c__Acidimicrobiia; o__Acidimicrobiales; f__wb1_P06; g__; s__ | 0 | N.D. | 69 | <1% |
| k__Bacteria; p__Actinobacteria; c__Actinobacteria; o__Actinomycetales; f__; g__; s__ | 5017 | 1% | 1447 | <1% |
| k__Bacteria; p__Actinobacteria; c__Actinobacteria; o__Actinomycetales; f__Actinomycetaceae; g__Actinobaculum; s__ | 1794 | <1% | 0 | N.D. |
| k__Bacteria; p__Actinobacteria; c__Actinobacteria; o__Actinomycetales; f__Actinomycetaceae; g__Actinobaculum; s__ | 1035 | <1% | 151 | <1% |
| k__Bacteria; p__Actinobacteria; c__Actinobacteria; o__Actinomycetales; f__Actinomycetaceae; g__Actinomyces; s__ | 785 | <1% | 0 | N.D. |
| k__Bacteria; p__Actinobacteria; c__Actinobacteria; o__Actinomycetales; f__Actinomycetaceae; g__Actinomyces; s__ | 826 | <1% | 0 | N.D. |
| k__Bacteria; p__Actinobacteria; c__Actinobacteria; o__Actinomycetales; f__Actinomycetaceae; g__Actinomyces; s__europaeus | 0 | N.D. | 552 | <1% |
| k__Bacteria; p__Actinobacteria; c__Actinobacteria; o__Actinomycetales; f__Actinomycetaceae; g__Trueperella; s__ | 1787 | <1% | 254 | <1% |
| k__Bacteria; p__Actinobacteria; c__Actinobacteria; o__Actinomycetales; f__Brevibacteriaceae; g__Brevibacterium; s__ | 3241 | 1% | 381 | <1% |
| k__Bacteria; p__Actinobacteria; c__Actinobacteria; o__Actinomycetales; f__Corynebacteriaceae; g__Corynebacterium; s__ | 704 | <1% | 5553 | 2% |
| k__Bacteria; p__Actinobacteria; c__Actinobacteria; o__Actinomycetales; f__Corynebacteriaceae; g__Corynebacterium; s__ | 8460 | 1% | 4053 | 1% |
| k__Bacteria; p__Actinobacteria; c__Actinobacteria; o__Actinomycetales; f__Corynebacteriaceae; g__Corynebacterium; s__ | 892 | <1% | 270 | <1% |
| k__Bacteria; p__Actinobacteria; c__Actinobacteria; o__Actinomycetales; f__Corynebacteriaceae; g__Corynebacterium; s__ | 497 | <1% | 670 | <1% |
| k__Bacteria; p__Actinobacteria; c__Actinobacteria; o__Actinomycetales; f__Corynebacteriaceae; g__Corynebacterium; s__ | 36215 | 6% | 3644 | 1% |
| k__Bacteria; p__Actinobacteria; c__Actinobacteria; o__Actinomycetales; f__Corynebacteriaceae; g__Corynebacterium; s__ | 7739 | 1% | 6 | <1% |
| k__Bacteria; p__Actinobacteria; c__Actinobacteria; o__Actinomycetales; f__Corynebacteriaceae; g__Corynebacterium; s__ | 5856 | 1% | 82 | <1% |

| K=kingdom, p=phyla, c=class, o=order, f=family, g=genus, s=species | total reads | Relative | total reads | Relative |
| --- | --- | --- | --- | --- |
|  | swabs | abundance | tissue | abundance |
| k__Bacteria; p__Actinobacteria; c__Actinobacteria; o__Actinomycetales; f__Corynebacteriaceae; g__Corynebacterium; s__simulans | 789 | <1% | 0 | N.D. |
| k__Bacteria; p__Actinobacteria; c__Actinobacteria; o__Actinomycetales; f__Dermabacteraceae; g__Dermabacter; s__ | 1565 | <1% | 2887 | 1% |
| k__Bacteria; p__Actinobacteria; c__Actinobacteria; o__Actinomycetales; f__Microbacteriaceae; g__Candidatus Rhodoluna; s__ | 104 | <1% | 0 | N.D. |
| k__Bacteria; p__Actinobacteria; c__Actinobacteria; o__Actinomycetales; f__Micromonosporaceae; g__; s__ | 0 | N.D. | 1059 | <1% |
| k__Bacteria; p__Actinobacteria; c__Actinobacteria; o__Actinomycetales; f__Propionibacteriaceae; g__Propionibacterium; s__acnes | 0 | N.D. | 504 | <1% |
| k__Bacteria; p__Actinobacteria; c__Coriobacteriia; o__Coriobacteriales; f__Coriobacteriaceae; g__Atopobium; s__ | 556 | <1% | 0 | N.D. |
| k__Bacteria; p__Actinobacteria; c__Actinobacteria; o__Actinomycetales; f__Pseudonocardiaceae; g__Prauserella; s__rugosa | 0 | N.D. | 104 | <1% |
| k__Bacteria; p__Aquificae; c__Aquificae; o__Aquificales; f__Aquificaceae; g__Hydrogenobaculum; s__ | 0 | N.D. | 1170 | <1% |
| k__Bacteria; p__Bacteroidetes; c__[Rhodothermi]; o__[Rhodothermales]; f__[Balneolaceae]; g__Balneola; s__ | 0 | N.D. | 188 | <1% |
| k__Bacteria; p__Bacteroidetes; c__[Rhodothermi]; o__[Rhodothermales]; f__Rhodothermaceae; g__; s__ | 0 | N.D. | 2077 | 1% |
| k__Bacteria; p__Bacteroidetes; c__[Saprospirae]; o__[Saprospirales]; f__Chitinophagaceae; g__Sediminibacterium; s__ | 0 | N.D. | 298 | <1% |
| k__Bacteria; p__Bacteroidetes; c__Bacteroidia; o__Bacteroidales; f__Bacteroidaceae; g__Bacteroides; s__ | 704 | <1% | 1854 | 1% |
| k__Bacteria; p__Bacteroidetes; c__Bacteroidia; o__Bacteroidales; f__Bacteroidaceae; g__Bacteroides; s__caccae | 1710 | <1% | 0 | N.D. |
| k__Bacteria; p__Bacteroidetes; c__Bacteroidia; o__Bacteroidales; f__Bacteroidaceae; g__Bacteroides; s__fragilis | 536 | <1% | 8657 | 3% |
| k__Bacteria; p__Bacteroidetes; c__Bacteroidia; o__Bacteroidales; f__Bacteroidaceae; g__Bacteroides; s__fragilis | 26880 | 4% | 10449 | 3% |
| k__Bacteria; p__Bacteroidetes; c__Bacteroidia; o__Bacteroidales; f__Porphyromonadaceae; g__Porphyromonas; s__ | 841 | <1% | 832 | <1% |
| k__Bacteria; p__Bacteroidetes; c__Bacteroidia; o__Bacteroidales; f__Porphyromonadaceae; g__Porphyromonas; s__ | 15259 | 2% | 2459 | 1% |
| k__Bacteria; p__Bacteroidetes; c__Bacteroidia; o__Bacteroidales; f__Porphyromonadaceae; g__Porphyromonas; s__ | 2754 | <1% | 349 | <1% |
| k__Bacteria; p__Bacteroidetes; c__Bacteroidia; o__Bacteroidales; f__Porphyromonadaceae; g__Porphyromonas; s__ | 5138 | 1% | 547 | <1% |
| k__Bacteria; p__Bacteroidetes; c__Bacteroidia; o__Bacteroidales; f__Prevotellaceae; g__Prevotella; s__ | 24639 | 4% | 7296 | 2% |
| k__Bacteria; p__Bacteroidetes; c__Bacteroidia; o__Bacteroidales; f__Prevotellaceae; g__Prevotella; s__ | 1512 | <1% | 1357 | <1% |
| k__Bacteria; p__Bacteroidetes; c__Bacteroidia; o__Bacteroidales; f__Prevotellaceae; g__Prevotella; s__ | 10420 | 2% | 3698 | 1% |
| k__Bacteria; p__Bacteroidetes; c__Bacteroidia; o__Bacteroidales; f__Prevotellaceae; g__Prevotella; s__ | 1199 | <1% | 7309 | 2% |
| k__Bacteria; p__Bacteroidetes; c__Bacteroidia; o__Bacteroidales; f__Prevotellaceae; g__Prevotella; s__ | 737 | <1% | 1329 | <1% |
| k__Bacteria; p__Bacteroidetes; c__Bacteroidia; o__Bacteroidales; f__Prevotellaceae; g__Prevotella; s__ | 23245 | 4% | 1230 | <1% |
| k__Bacteria; p__Bacteroidetes; c__Bacteroidia; o__Bacteroidales; f__Prevotellaceae; g__Prevotella; s__ | 1185 | <1% | 746 | <1% |

| K=kingdom, p=phyla, c=class, o=order, f=family, g=genus, s=species | total reads | Relative | total reads | Relative |
| --- | --- | --- | --- | --- |
|  | swabs | abundance | tissue | abundance |
| k__Bacteria; p__Bacteroidetes; c__Bacteroidia; o__Bacteroidales; f__Prevotellaceae; g__Prevotella; s__ | 0 | N.D. | 443 | <1% |
| k__Bacteria; p__Bacteroidetes; c__Bacteroidia; o__Bacteroidales; f__Prevotellaceae; g__Prevotella; s__ | 0 | N.D. | 819 | <1% |
| k__Bacteria; p__Bacteroidetes; c__Bacteroidia; o__Bacteroidales; f__Prevotellaceae; g__Prevotella; s__melaninogenica | 12591 | 2% | 3594 | 1% |
| k__Bacteria; p__Bacteroidetes; c__Bacteroidia; o__Bacteroidales; f__Prevotellaceae; g__Prevotella; s__ | 0 | N.D. | 2874 | 1% |
| k__Bacteria; p__Bacteroidetes; c__Flavobacteriia; o__Flavobacteriales; f__[Weeksellaceae]; g__Elizabethkingia; s__meningoseptica | 0 | N.D. | 1204 | <1% |
| k__Bacteria; p__Bacteroidetes; c__Flavobacteriia; o__Flavobacteriales; f__Flavobacteriaceae; g__Flavobacterium; s__ | 176 | <1% | 638 | <1% |
| k__Bacteria; p__Cyanobacteria; c__Chloroplast; o__Chlorophyta; f__Ulvophyceae; g__; s__ | 543 | <1% | 0 | N.D. |
| k__Bacteria; p__Chlorobi; c__Chlorobia; o__Chlorobiales; f__Chlorobiaceae; g__; s__ | 891 | <1% | 1696 | 1% |
| k__Bacteria; p__Chlorobi; c__Chlorobia; o__Chlorobiales; f__Chlorobiaceae; g__Chlorobaculum; s__ | 719 | <1% | 638 | <1% |
| k__Bacteria; p__Chloroflexi; c__Chloroflexi; o__Herpetosiphonales; f__; g__; s__ | 0 | N.D. | 1446 | <1% |
| k__Bacteria; p__Chloroflexi; c__SAR202; o__; f__; g__; s__ | 0 | N.D. | 277 | <1% |
| k__Bacteria; p__Cyanobacteria; c__Chloroplast; o__Streptophyta; f__; g__; s__ | 0 | N.D. | 870 | <1% |
| k__Bacteria; p__Cyanobacteria; c__Nostocophycideae; o__Nostocales; f__Nostocaceae; g__; s__ | 0 | N.D. | 345 | <1% |
| k__Bacteria; p__Cyanobacteria; c__Synechococcophycideae; o__Synechococcales; f__Synechococcaceae; g__Synechococcus; s__ | 0 | N.D. | 2701 | 1% |
| k__Bacteria; p__Firmicutes; c__Bacilli; o__Bacillales; f__Staphylococcaceae; g__Jeotgalicoccus; s__ | 0 | N.D. | 0 | N.D. |
| k__Bacteria; p__Firmicutes; c__Bacilli; o__Bacillales; f__Staphylococcaceae; g__Jeotgalicoccus; s__ | 0 | N.D. | 0 | N.D. |
| k__Bacteria; p__Firmicutes; c__Bacilli; o__Bacillales; f__Staphylococcaceae; g__Staphylococcus; s__ | 3545 | 1% | 0 | N.D. |
| k__Bacteria; p__Firmicutes; c__Bacilli; o__Bacillales; f__Staphylococcaceae; g__Staphylococcus; s__ | 16504 | 3% | 2937 | 1% |
| k__Bacteria; p__Firmicutes; c__Bacilli; o__Bacillales; f__Staphylococcaceae; g__Staphylococcus; s__ | 431 | <1% | 2967 | 1% |
| k__Bacteria; p__Firmicutes; c__Bacilli; o__Bacillales; f__Staphylococcaceae; g__Staphylococcus; s__aureus | 330 | <1% | 2613 | 1% |
| k__Bacteria; p__Firmicutes; c__Bacilli; o__Bacillales; f__Staphylococcaceae; g__Staphylococcus; s__aureus | 91101 | 14% | 2673 | 1% |
| k__Bacteria; p__Firmicutes; c__Bacilli; o__Bacillales; f__Staphylococcaceae; g__Staphylococcus; s__sciuri | 926 | <1% | 0 | N.D. |
| k__Bacteria; p__Firmicutes; c__Bacilli; o__Gemellales; f__Gemellaceae; g__Gemella; s__ | 241 | <1% | 355 | <1% |
| k__Bacteria; p__Firmicutes; c__Bacilli; o__Lactobacillales; f__Enterococcaceae; g__Enterococcus; s__ | 2273 | <1% | 11223 | 4% |
| k__Bacteria; p__Firmicutes; c__Bacilli; o__Lactobacillales; f__Streptococcaceae; g__Streptococcus; s__ | 30489 | 5% | 27048 | 9% |
| k__Bacteria; p__Firmicutes; c__Bacilli; o__Lactobacillales; f__Streptococcaceae; g__Streptococcus; s__infantis | 650 | <1% | 3634 | 1% |

| K=kingdom, p=phyla, c=class, o=order, f=family, g=genus, s=species | total reads | Relative | total reads | Relative |
| --- | --- | --- | --- | --- |
|  | swabs | abundance | tissue | abundance |
| k__Bacteria; p__Firmicutes; c__Bacilli; o__Lactobacillales; f__Streptococcaceae; g__Streptococcus; s__infantis | 19486 | 3% | 0 | N.D. |
| k__Bacteria; p__Firmicutes; c__Clostridia; o__Clostridiales; f__[Tissierellaceae]; g__; s__ | 3008 | <1% | 326 | <1% |
| k__Bacteria; p__Firmicutes; c__Clostridia; o__Clostridiales; f__[Tissierellaceae]; g__1-68; s__ | 0 | N.D. | 405 | <1% |
| k__Bacteria; p__Firmicutes; c__Clostridia; o__Clostridiales; f__[Tissierellaceae]; g__Anaerococcus; s__ | 1721 | <1% | 9809 | 3% |
| k__Bacteria; p__Firmicutes; c__Clostridia; o__Clostridiales; f__[Tissierellaceae]; g__Anaerococcus; s__ | 21877 | 3% | 948 | <1% |
| k__Bacteria; p__Firmicutes; c__Clostridia; o__Clostridiales; f__[Tissierellaceae]; g__Anaerococcus; s__ | 400 | <1% | 700 | <1% |
| k__Bacteria; p__Firmicutes; c__Clostridia; o__Clostridiales; f__[Tissierellaceae]; g__Anaerococcus; s__ | 5096 | 1% | 1386 | <1% |
| k__Bacteria; p__Firmicutes; c__Clostridia; o__Clostridiales; f__[Tissierellaceae]; g__Anaerococcus; s__ | 1391 | <1% | 145 | <1% |
| k__Bacteria; p__Firmicutes; c__Clostridia; o__Clostridiales; f__[Tissierellaceae]; g__Anaerococcus; s__ | 24184 | 4% | 710 | <1% |
| k__Bacteria; p__Firmicutes; c__Clostridia; o__Clostridiales; f__[Tissierellaceae]; g__Anaerococcus; s__ | 416 | <1% | 152 | <1% |
| k__Bacteria; p__Firmicutes; c__Clostridia; o__Clostridiales; f__[Tissierellaceae]; g__Anaerococcus; s__ | 0 | N.D. | 4845 | 2% |
| k__Bacteria; p__Firmicutes; c__Clostridia; o__Clostridiales; f__[Tissierellaceae]; g__Finegoldia; s__ | 0 | N.D. | 5331 | 2% |
| k__Bacteria; p__Firmicutes; c__Clostridia; o__Clostridiales; f__[Tissierellaceae]; g__Finegoldia; s__ | 13275 | 2% | 10540 | 3% |
| k__Bacteria; p__Firmicutes; c__Clostridia; o__Clostridiales; f__[Tissierellaceae]; g__Gallicola; s__ | 0 | N.D. | 70 | <1% |
| k__Bacteria; p__Firmicutes; c__Clostridia; o__Clostridiales; f__[Tissierellaceae]; g__Helcococcus; s__ | 2533 | <1% | 0 | N.D. |
| k__Bacteria; p__Firmicutes; c__Clostridia; o__Clostridiales; f__[Tissierellaceae]; g__Helcococcus; s__ | 1440 | <1% | 1219 | <1% |
| k__Bacteria; p__Firmicutes; c__Clostridia; o__Clostridiales; f__[Tissierellaceae]; g__Helcococcus; s__ | 0 | N.D. | 79 | <1% |
| k__Bacteria; p__Firmicutes; c__Clostridia; o__Clostridiales; f__[Tissierellaceae]; g__Peptoniphilus; s__ | 0 | N.D. | 440 | <1% |
| k__Bacteria; p__Firmicutes; c__Clostridia; o__Clostridiales; f__[Tissierellaceae]; g__Peptoniphilus; s__ | 0 | N.D. | 6483 | 2% |
| k__Bacteria; p__Firmicutes; c__Clostridia; o__Clostridiales; f__[Tissierellaceae]; g__Peptoniphilus; s__ | 0 | N.D. | 4405 | 1% |
| k__Bacteria; p__Firmicutes; c__Clostridia; o__Clostridiales; f__[Tissierellaceae]; g__Peptoniphilus; s__ | 528 | <1% | 148 | <1% |
| k__Bacteria; p__Firmicutes; c__Clostridia; o__Clostridiales; f__[Tissierellaceae]; g__Peptoniphilus; s__ | 2491 | <1% | 1504 | <1% |
| k__Bacteria; p__Firmicutes; c__Clostridia; o__Clostridiales; f__[Tissierellaceae]; g__Peptoniphilus; s__ | 2860 | <1% | 74 | <1% |
| k__Bacteria; p__Firmicutes; c__Clostridia; o__Clostridiales; f__[Tissierellaceae]; g__Peptoniphilus; s__ | 313 | <1% | 6686 | 2% |
| k__Bacteria; p__Firmicutes; c__Clostridia; o__Clostridiales; f__[Tissierellaceae]; g__Peptoniphilus; s__ | 370 | <1% | 550 | <1% |
| k__Bacteria; p__Firmicutes; c__Clostridia; o__Clostridiales; f__[Tissierellaceae]; g__Peptoniphilus; s__ | 15386 | 2% | 348 | <1% |

| K=kingdom, p=phyla, c=class, o=order, f=family, g=genus, s=species | total reads | Relative | total reads | Relative |
| --- | --- | --- | --- | --- |
|  | swabs | abundance | tissue | abundance |
| k__Bacteria; p__Firmicutes; c__Clostridia; o__Clostridiales; f__[Tissierellaceae]; g__Peptoniphilus; s__ | 402 | <1% | 0 | N.D. |
| k__Bacteria; p__Firmicutes; c__Clostridia; o__Clostridiales; f__[Tissierellaceae]; g__WAL_1855D; s__ | 0 | N.D. | 92 | <1% |
| k__Bacteria; p__Firmicutes; c__Clostridia; o__Clostridiales; f__Clostridiaceae; g__Clostridium; s__ | 1581 | <1% | 489 | <1% |
| k__Bacteria; p__Firmicutes; c__Clostridia; o__Clostridiales; f__Lachnospiraceae; g__; s__ | 25 | <1% | 4 | <1% |
| k__Bacteria; p__Firmicutes; c__Clostridia; o__Clostridiales; f__Peptococcaceae; g__Peptococcus; s__ | 0 | N.D. | 489 | <1% |
| k__Bacteria; p__Firmicutes; c__Clostridia; o__Clostridiales; f__Peptostreptococcaceae; g__Peptostreptococcus; s__anaerobius | 19743 | 3% | 2319 | 1% |
| k__Bacteria; p__Firmicutes; c__Clostridia; o__Clostridiales; f__Ruminococcaceae; g__; s__ | 464 | <1% | 0 | N.D. |
| k__Bacteria; p__Firmicutes; c__Clostridia; o__Clostridiales; f__Veillonellaceae; g__Dialister; s__ | 760 | <1% | 0 | N.D. |
| k__Bacteria; p__Firmicutes; c__Clostridia; o__Clostridiales; f__Veillonellaceae; g__Veillonella; s__dispar | 2546 | <1% | 172 | <1% |
| k__Bacteria; p__Firmicutes; c__Clostridia; o__Clostridiales; f__Veillonellaceae; g__Veillonella; s__dispar | 6250 | 1% | 589 | <1% |
| k__Bacteria; p__Firmicutes; c__Clostridia; o__Clostridiales; f__Veillonellaceae; g__Veillonella; s__dispar | 2353 | <1% | 0 | N.D. |
| k__Bacteria; p__Firmicutes; c__Clostridia; o__Clostridiales; f__Veillonellaceae; g__Veillonella; s__dispar | 410 | <1% | 0 | N.D. |
| k__Bacteria; p__Firmicutes; c__Clostridia; o__Clostridiales; f__Veillonellaceae; g__Veillonella; s__parvula | 47 | <1% | 0 | N.D. |
| k__Bacteria; p__Firmicutes; c__Clostridia; o__Thermoanaerobacterales; f__Caldicellulosiruptoraceae; g__Caldicellulosiruptor; s__saccharolyticus | 0 | N.D. | 345 | <1% |
| k__Bacteria; p__Firmicutes; c__Clostridia; o__Thermoanaerobacterales; f__Thermoanaerobacteraceae; g__Thermoanaerobacter; s__ | 0 | N.D. | 1230 | <1% |
| k__Bacteria; p__Firmicutes; c__Clostridia; o__Thermoanaerobacterales; f__Thermoanaerobacteraceae; g__Thermoanaerobacter; s__ | 0 | N.D. | 470 | <1% |
| k__Bacteria; p__Firmicutes; c__Erysipelotrichi; o__Erysipelotrichales; f__Erysipelotrichaceae; g__; s__ | 0 | N.D. | 537 | <1% |
| k__Bacteria; p__Nitrospirae; c__Nitrospira; o__Nitrospirales; f__Nitrospiraceae; g__; s__ | 0 | N.D. | 129 | <1% |
| k__Bacteria; p__Planctomycetes; c__Planctomycetia; o__Planctomycetales; f__Planctomycetaceae; g__Planctomyces; s__ | 0 | N.D. | 167 | <1% |
| k__Bacteria; p__Fusobacteria; c__Fusobacteriia; o__Fusobacteriales; f__Fusobacteriaceae; g__Fusobacterium; s__ | 3007 | <1% | 0 | N.D. |
| k__Bacteria; p__Fusobacteria; c__Fusobacteriia; o__Fusobacteriales; f__Fusobacteriaceae; g__Fusobacterium; s__ | 318 | <1% | 0 | N.D. |
| k__Bacteria; p__Fusobacteria; c__Fusobacteriia; o__Fusobacteriales; f__Fusobacteriaceae; g__Fusobacterium; s__ | 100 | <1% | 0 | N.D. |
| k__Bacteria; p__Fusobacteria; c__Fusobacteriia; o__Fusobacteriales; f__Fusobacteriaceae; g__Fusobacterium; s__ | 5076 | 1% | 0 | N.D. |
| k__Bacteria; p__Fusobacteria; c__Fusobacteriia; o__Fusobacteriales; f__Fusobacteriaceae; g__Fusobacterium; s__ | 140 | <1% | 542 | <1% |
| k__Bacteria; p__Gemmatimonadetes; c__Gemm-2; o__; f__; g__; s__ | 0 | N.D. | 46 | <1% |
| k__Bacteria; p__Nitrospirae; c__Nitrospira; o__Nitrospirales; f__Nitrospiraceae; g__; s__ | 0 | N.D. | 23 | <1% |

| K=kingdom, p=phyla, c=class, o=order, f=family, g=genus, s=species | total reads | Relative | total reads | Relative |
| --- | --- | --- | --- | --- |
|  | swabs | abundance | tissue | abundance |
| k__Bacteria; p__Planctomycetes; c__Planctomycetia; o__Planctomycetales; f__Planctomycetaceae; g__Planctomyces; s__ | 0 | N.D. | 58 | <1% |
| k__Bacteria; p__Proteobacteria; c__Alphaproteobacteria; o__Rhizobiales; f__; g__; s__ | 0 | N.D. | 319 | <1% |
| k__Bacteria; p__Proteobacteria; c__Alphaproteobacteria; o__Caulobacterales; f__Caulobacteraceae; g__Brevundimonas; s__diminuta | 512 | <1% | 0 | N.D. |
| k__Bacteria; p__Proteobacteria; c__Alphaproteobacteria; o__Rhodobacterales; f__Rhodobacteraceae; g__; s__ | 0 | N.D. | 15 | <1% |
| k__Bacteria; p__Proteobacteria; c__Alphaproteobacteria; o__Rhodobacterales; f__Rhodobacteraceae; g__; s__ | 0 | N.D. | 110 | <1% |
| k__Bacteria; p__Proteobacteria; c__Alphaproteobacteria; o__Rhodobacterales; f__Rhodobacteraceae; g__; s__ | 0 | N.D. | 15 | <1% |
| k__Bacteria; p__Proteobacteria; c__Alphaproteobacteria; o__Rhodobacterales; f__Rhodobacteraceae; g__; s__ | 0 | N.D. | 156 | <1% |
| k__Bacteria; p__Proteobacteria; c__Alphaproteobacteria; o__Rhodobacterales; f__Rhodobacteraceae; g__; s__ | 0 | N.D. | 497 | <1% |
| k__Bacteria; p__Proteobacteria; c__Alphaproteobacteria; o__Rhodobacterales; f__Rhodobacteraceae; g__; s__ | 0 | N.D. | 358 | <1% |
| k__Bacteria; p__Proteobacteria; c__Alphaproteobacteria; o__Rhodobacterales; f__Rhodobacteraceae; g__Rhodobaca; s__ | 0 | N.D. | 330 | <1% |
| k__Bacteria; p__Proteobacteria; c__Alphaproteobacteria; o__Rhodobacterales; f__Rhodobacteraceae; g__Rhodobacter; s__ | 0 | N.D. | 32 | <1% |
| k__Bacteria; p__Proteobacteria; c__Alphaproteobacteria; o__Rickettsiales; f__mitochondria; g__; s__ | 0 | N.D. | 401 | <1% |
| k__Bacteria; p__Proteobacteria; c__Alphaproteobacteria; o__Rickettsiales; f__Rickettsiaceae; g__Wolbachia; s__ | 0 | N.D. | 94 | <1% |
| k__Bacteria; p__Proteobacteria; c__Betaproteobacteria; o__Burkholderiales; f__Alcaligenaceae; g__; s__ | 6726 | 1% | 116 | <1% |
| k__Bacteria; p__Proteobacteria; c__Betaproteobacteria; o__Burkholderiales; f__Comamonadaceae; g__Delftia; s__ | 0 | N.D. | 238 | <1% |
| k__Bacteria; p__Proteobacteria; c__Betaproteobacteria; o__Burkholderiales; f__Comamonadaceae; g__Pelomonas; s__puraquae | 0 | N.D. | 314 | <1% |
| k__Bacteria; p__Proteobacteria; c__Betaproteobacteria; o__Neisseriales; f__Neisseriaceae; g__Eikenella; s__ | 746 | 0% | 0 | N.D. |
| k__Bacteria; p__Proteobacteria; c__Betaproteobacteria; o__Nitrosomonadales; f__Nitrosomonadaceae; g__; s__ | 0 | N.D. | 382 | <1% |
| k__Bacteria; p__Proteobacteria; c__Deltaproteobacteria; o__Myxococcales; f__; g__; s__ | 0 | N.D. | 251 | <1% |
| k__Bacteria; p__Proteobacteria; c__Deltaproteobacteria; o__NB1-j; f__NB1-i; g__; s__ | 0 | N.D. | 2389 | 1% |
| k__Bacteria; p__Proteobacteria; c__Epsilonproteobacteria; o__Campylobacterales; f__Campylobacteraceae; g__Arcobacter; s__ | 0 | N.D. | 153 | <1% |
| k__Bacteria; p__Proteobacteria; c__Epsilonproteobacteria; o__Campylobacterales; f__Campylobacteraceae; g__Arcobacter; s__ | 0 | N.D. | 356 | <1% |
| k__Bacteria; p__Proteobacteria; c__Epsilonproteobacteria; o__Campylobacterales; f__Campylobacteraceae; g__Campylobacter; s__ureolyticus | 1042 | <1% | 914 | <1% |
| k__Bacteria; p__Proteobacteria; c__Gammaproteobacteria; o__Aeromonadales; f__Aeromonadaceae; g__; s__ | 1779 | <1% | 0 | N.D. |
| k__Bacteria; p__Proteobacteria; c__Gammaproteobacteria; o__Alteromonadales; f__HTCC2188; g__HTCC; s__ | 0 | N.D. | 221 | <1% |
| k__Bacteria; p__Proteobacteria; c__Gammaproteobacteria; o__Alteromonadales; f__HTCC2188; g__HTCC; s__ | 0 | N.D. | 487 | <1% |

| K=kingdom, p=phyla, c=class, o=order, f=family, g=genus, s=species | total reads | Relative | total reads | Relative |
| --- | --- | --- | --- | --- |
|  | swabs | abundance | tissue | abundance |
| k__Bacteria; p__Proteobacteria; c__Gammaproteobacteria; o__Alteromonadales; f__Shewanellaceae; g__Shewanella; s__ | 0 | N.D. | 1159 | <1% |
| k__Bacteria; p__Proteobacteria; c__Gammaproteobacteria; o__Chromatiales; f__; g__; s__ | 0 | N.D. | 327 | <1% |
| k__Bacteria; p__Proteobacteria; c__Gammaproteobacteria; o__Chromatiales; f__; g__; s__ | 0 | N.D. | 233 | <1% |
| k__Bacteria; p__Proteobacteria; c__Gammaproteobacteria; o__Chromatiales; f__Ectothiorhodospiraceae; g__; s__ | 0 | N.D. | 140 | <1% |
| k__Bacteria; p__Proteobacteria; c__Gammaproteobacteria; o__Enterobacteriales; f__Enterobacteriaceae; g__; s__ | 548 | <1% | 308 | <1% |
| k__Bacteria; p__Proteobacteria; c__Gammaproteobacteria; o__Enterobacteriales; f__Enterobacteriaceae; g__; s__ | 27914 | 4% | 1587 | 1% |
| k__Bacteria; p__Proteobacteria; c__Gammaproteobacteria; o__Enterobacteriales; f__Enterobacteriaceae; g__; s__ | 1271 | <1% | 17 | <1% |
| k__Bacteria; p__Proteobacteria; c__Gammaproteobacteria; o__Enterobacteriales; f__Enterobacteriaceae; g__; s__ | 2678 | <1% | 12564 | 4% |
| k__Bacteria; p__Proteobacteria; c__Gammaproteobacteria; o__Enterobacteriales; f__Enterobacteriaceae; g__Erwinia; s__ | 0 | N.D. | 111 | <1% |
| k__Bacteria; p__Proteobacteria; c__Gammaproteobacteria; o__Enterobacteriales; f__Enterobacteriaceae; g__Klebsiella; s__ | 0 | N.D. | 140 | <1% |
| k__Bacteria; p__Proteobacteria; c__Gammaproteobacteria; o__Enterobacteriales; f__Enterobacteriaceae; g__Klebsiella; s__ | 0 | N.D. | 102 | <1% |
| k__Bacteria; p__Proteobacteria; c__Gammaproteobacteria; o__Enterobacteriales; f__Enterobacteriaceae; g__Morganella; s__ | 546 | <1% | 0 | N.D. |
| k__Bacteria; p__Proteobacteria; c__Gammaproteobacteria; o__Enterobacteriales; f__Enterobacteriaceae; g__Proteus; s__ | 0 | N.D. | 561 | <1% |
| k__Bacteria; p__Proteobacteria; c__Gammaproteobacteria; o__Enterobacteriales; f__Enterobacteriaceae; g__Proteus; s__ | 17459 | 3% | 12096 | <1% |
| k__Bacteria; p__Proteobacteria; c__Gammaproteobacteria; o__Enterobacteriales; f__Enterobacteriaceae; g__Providencia; s__ | 706 | <1% | 0 | N.D. |
| k__Bacteria; p__Proteobacteria; c__Gammaproteobacteria; o__Enterobacteriales; f__Enterobacteriaceae; g__Serratia; s__ | 6759 | 1% | 1225 | <1% |
| k__Bacteria; p__Proteobacteria; c__Gammaproteobacteria; o__Enterobacteriales; f__Enterobacteriaceae; g__Serratia; s__marcescens | 36543 | 6% | 7375 | 2% |
| k__Bacteria; p__Proteobacteria; c__Gammaproteobacteria; o__HTCC2188; f__HTCC2089; g__; s__ | 0 | N.D. | 382 | <1% |
| k__Bacteria; p__Proteobacteria; c__Gammaproteobacteria; o__HTCC2188; f__HTCC2089; g__; s__ | 0 | N.D. | 190 | <1% |
| k__Bacteria; p__Proteobacteria; c__Gammaproteobacteria; o__Pasteurellales; f__Pasteurellaceae; g__Haemophilus; s__parainfluenzae | 2302 | <1% | 1294 | <1% |
| k__Bacteria; p__Proteobacteria; c__Gammaproteobacteria; o__Pseudomonadales; f__Moraxellaceae; g__Acinetobacter; s__lwoffii | 0 | N.D. | 59 | <1% |
| k__Bacteria; p__Proteobacteria; c__Gammaproteobacteria; o__Pseudomonadales; f__Pseudomonadaceae; g__Pseudomonas; s__ | 16500 | 3% | 33657 | 11% |
| k__Bacteria; p__Proteobacteria; c__Gammaproteobacteria; o__Pseudomonadales; f__Pseudomonadaceae; g__Pseudomonas; s__stutzeri | 0 | N.D. | 1 | <1% |
| k__Bacteria; p__Proteobacteria; c__Gammaproteobacteria; o__Thiohalorhabdales; f__; g__; s__ | 0 | N.D. | 353 | <1% |
| k__Bacteria; p__Proteobacteria; c__Gammaproteobacteria; o__Thiohalorhabdales; f__; g__; s__ | 0 | N.D. | 244 | <1% |
| k__Bacteria; p__Proteobacteria; c__Gammaproteobacteria; o__Thiohalorhabdales; f__; g__; s__ | 0 | N.D. | 3098 | 1% |

| K=kingdom, p=phyla, c=class, o=order, f=family, g=genus, s=species | total reads | Relative | total reads | Relative |
| --- | --- | --- | --- | --- |
|  | swabs | abundance | tissue | abundance |
| k__Bacteria; p__Proteobacteria; c__Gammaproteobacteria; o__Thiohalorhabdales; f__; g__; s__ | 0 | N.D. | 182 | <1% |
| k__Bacteria; p__Proteobacteria; c__Gammaproteobacteria; o__Thiotrichales; f__Piscirickettsiaceae; g__; s__ | 0 | N.D. | 567 | <1% |
| k__Bacteria; p__Proteobacteria; c__Gammaproteobacteria; o__Xanthomonadales; f__Xanthomonadaceae; g__Stenotrophomonas; s__geniculata | 215 | <1% | 815 | <1% |
| k__Bacteria; p__SBR1093; c__EC214; o__; f__; g__; s__ | 0 | N.D. | 295 | <1% |
| k__Bacteria; p__Thermotogae; c__Thermotogae; o__Thermotogales; f__Thermotogaceae; g__Thermotoga; s__ | 0 | N.D. | 448 | <1% |
| **Total no. reads**  **Total number of reads**  **N.D. = not detected** | **640274** |  | **305110** |  |

N.D. =not detected
