# Supplementary material for: The microbiome of diabetic foot ulcers: a comparison of swab and tissue biopsy wound sampling techniques using 16S rRNA gene sequencing
Source: BMC Microbiol. 2020 Jun 16;20:163. doi: 10.1186/s12866-020-01843-2 (PMC7296698; doi:10.1186/s12866-020-01843-2)
Supplement: Supplementary file 3 — Additional file 3 Supplementary Table S5. Dendogram from hierarchical cluster analysis of bacterial communities indicating no clustering [file 12866_2020_1843_MOESM3_ESM.docx]

## Supplementary Table S5 Additional File 3

**Dendogram from hierarchical cluster analysis of bacterial communities indicating no clustering**
