# Supplementary material for: The microbiome of diabetic foot ulcers: a comparison of swab and tissue biopsy wound sampling techniques using 16S rRNA gene sequencing
Source: BMC Microbiol. 2020 Jun 16;20:163. doi: 10.1186/s12866-020-01843-2 (PMC7296698; doi:10.1186/s12866-020-01843-2)
Supplement: Supplementary file 4 — Additional file 4 Supplementary Table S6. Demographics of 20 participants [file 12866_2020_1843_MOESM4_ESM.docx]

**Supplementary Table S6 Additional File 4**

**Demographics of 20 participants**

**IDSA=Infectious Disease Society of America**

| **Participant** | **Age** | **Gender** | **Type of Diabetes** | **Duration** | **Insulin** | **Ulcer site** | **Length** | **Depth** | **Granulation** | **HbA1c** | **Duration of** | **IDSA score (1=uninfected** |
| --- | --- | --- | --- | --- | --- | --- | --- | --- | --- | --- | --- | --- |
| **number** |  |  | **1 or 2** | **(years)** |  |  | **x width( mm)** | **(mm)** |  |  | **Ulcer (weeks)** | **2=mild,3=mod,4=severe)** |
| **1** | 63 | MALE | 2 | 12 | N | RIGHT DORSAL 1ST MTPF | 40X30 | 30 | 20 | 6.2 | 26 | 4 |
| **2** | 53 | MALE | 2 | 15 | Y | LEFT PLANTAR 3 MET HEAD | 40X20 | 5 | 100 | 8.1 | 32 | 2 |
| **3** | 64 | MALE | 2 | 10 | N | LEFT HALLUX APEX | 20X10 | 10 | 90 | 10.2 | 12 | 4 |
| **4** | 78 | MALE | 2 | 16 | N | RIGHT MEDIAL HALLUX | 30X30 | 40 | 0 | 6.2 | 8 | 3 |
| **5** | 79 | MALE | 2 | 15 | N | LEFT HALLUX APEX | 10X10 | 0 | 100 | 7.2 | 9 | 2 |
| **6** | 37 | MALE | 2 | 8 | N | LEFT PLANTAR 2 MET HEAD | 10X10 | 10 | 70 | 7 | 6 | 3 |
| **7** | 49 | FEMALE | 2 | 10 | N | LEFT PLANTAR MIDFOOT | 60X40 | 10 | 80 |  | 52 | 4 |
| **8** | 72 | MALE | 2 | 7 | N | LEFT RETRO CALCANEUM | 55X30 | 0 | 70 | 6.7 | 72 | 1 |
| **9** | 71 | MALE | 2 | 1 | N | LEFT DORSUM | 50X30 | 20 | 40 | N/A | 6 | 1 |
| **10** | 58 | MALE | 2 | 10 | Y | LEFT PLANTAR 5TH MET HEAD | 18X31 | 15 | 100 | N/A | 24 | 1 |
| **11** | 67 | MALE | 2 | 13 | Y | LEFT 1ST PLANTAR MET HEAD | 45X25 | 10 | 90 | N/A | 12 | 1 |
| **12** | 51 | FEMALE | 2 | 22 | N | LEFT PLANTAR CALCANEUS | 40X50 | 0 | 0 | N/A | 7 | 1 |
| **13** | 62 | FEMALE | 2 | 22 | Y | LEFT PLANTAR MIDFOOT | 20X20 | 10 | 100 | N/A | 28 | 1 |
| **14** | 30 | FEMALE | 1 | 15 | Y | RIGHT PLANTAR 5TH MET HEAD | 20X20 | 5 | 100 | N/A | 27 | 1 |
| **15** | 63 | MALE | 1 | 20 | N | LEFT PLANTAR 1ST MET HEAD | 25X40 | 5 | 100 | N/A | 28 | 1 |
| **16** | 53 | MALE | 2 | 10 | N | LEFT 1ST HALLUX STUMP | 50X20 | 40 | 50 | N/A | 6 | 1 |
| **17** | 51 | MALE | 2 | 18 | N | LEFT PLANTAR HEEL | 50X53 | 10 | 80 | N/A | 6 | 1 |
| **18** | 78 | MALE | 2 | 16 | N | LEFT ACHILLES TENDON | 40X25 | 0 | 50 | N/A | 20 | 1 |
| **19** | 61 | MALE | 2 | 8 | N | RIGHT LATERAL HEEL ULCER | 40X25 | 5 | 60 | N/A | 10 | 1 |
| **20** | 41 | MALE | 2 | 3 | N | DORSAL RIGHT FOOT | 80X30 | 0 | 90 | N/A | 8 | 2 |
