# Supplementary material for: The microbiome of diabetic foot ulcers: a comparison of swab and tissue biopsy wound sampling techniques using 16S rRNA gene sequencing
Source: BMC Microbiol. 2020 Jun 16;20:163. doi: 10.1186/s12866-020-01843-2 (PMC7296698; doi:10.1186/s12866-020-01843-2)
Supplement: Supplementary file 5 — Additional file 5 Supplementary Table S7. Positive control data (tissue biopsies) using tissue biopsy DNA extraction method. Supplementary Table S8. Positive control data (swabs). Supplementary Table S9. Negative control data (swabs). Supplementary Table S10. Copan swab control data. Supplementary Table S11. Quantitative PCR data for positive and negative swab controls [file 12866_2020_1843_MOESM5_ESM.docx]

**Supplementary Table S7 Additional File 5**

**Positive Control data (tissue biopsies)** using tissue biopsy DNA extraction method

**A**=*Staphylococcus aureus* and *Acinetobacter rhizosphaerae* 1:5 **B**= *S.aureus* and *A.rhizosphaerae* 5:1

| Organism | A. | B. |
| --- | --- | --- |
| k__Archaea; p__[Parvarchaeota]; c__[Micrarchaea]; o__[Micrarchaeles]; f__; g__; s__ | 0 | 1 |
| k__Archaea; p__[Parvarchaeota]; c__[Micrarchaea]; o__[Micrarchaeles]; f__; g__; s__ | 0 | 0 |
| k__Bacteria; p__Acidobacteria; c__Acidobacteriia; o__Acidobacteriales; f__Acidobacteriaceae; g__; s__ | 0 | 0 |
| k__Bacteria; p__Actinobacteria; c__Actinobacteria; o__Actinomycetales; f__; g__; s__ | 0 | 0 |
| k__Bacteria; p__Actinobacteria; c__Actinobacteria; o__Actinomycetales; f__Actinomycetaceae; g__Actinomyces; s__ | 0 | 0 |
| k__Bacteria; p__Actinobacteria; c__Actinobacteria; o__Actinomycetales; f__Actinomycetaceae; g__Actinomyces; s__europaeus | 0 | 0 |
| k__Bacteria; p__Actinobacteria; c__Actinobacteria; o__Actinomycetales; f__Actinomycetaceae; g__Trueperella; s__ | 0 | 0 |
| k__Bacteria; p__Actinobacteria; c__Actinobacteria; o__Actinomycetales; f__Actinomycetaceae; g__Trueperella; s__ | 0 | 0 |
| Organism | A. | B. |
| k__Bacteria; p__Actinobacteria; c__Actinobacteria; o__Actinomycetales; f__Brevibacteriaceae; g__Brevibacterium; s__ | 0 | 0 |
| k__Bacteria; p__Actinobacteria; c__Actinobacteria; o__Actinomycetales; f__Corynebacteriaceae; g__Corynebacterium; s__ | 0 | 0 |
| k__Bacteria; p__Actinobacteria; c__Actinobacteria; o__Actinomycetales; f__Corynebacteriaceae; g__Corynebacterium; s__ | 0 | 0 |
| k__Bacteria; p__Actinobacteria; c__Actinobacteria; o__Actinomycetales; f__Corynebacteriaceae; g__Corynebacterium; s__ | 0 | 0 |
| k__Bacteria; p__Actinobacteria; c__Actinobacteria; o__Actinomycetales; f__Corynebacteriaceae; g__Corynebacterium; s__ | 0 | 0 |
| k__Bacteria; p__Actinobacteria; c__Actinobacteria; o__Actinomycetales; f__Corynebacteriaceae; g__Corynebacterium; s__ | 0 | 0 |
| k__Bacteria; p__Actinobacteria; c__Actinobacteria; o__Actinomycetales; f__Corynebacteriaceae; g__Corynebacterium; s__ | 0 | 0 |
| k__Bacteria; p__Actinobacteria; c__Actinobacteria; o__Actinomycetales; f__Corynebacteriaceae; g__Corynebacterium; s__kroppenstedtii | 0 | 0 |
| k__Bacteria; p__Actinobacteria; c__Actinobacteria; o__Actinomycetales; f__Corynebacteriaceae; g__Corynebacterium; s__simulans | 0 | 0 |
| k__Bacteria; p__Actinobacteria; c__Actinobacteria; o__Actinomycetales; f__Dermabacteraceae; g__Dermabacter; s__ | 0 | 0 |
| k__Bacteria; p__Actinobacteria; c__Actinobacteria; o__Actinomycetales; f__Micrococcaceae; g__Micrococcus; s__luteus | 0 | 0 |
| Organism | A. | B. |
| k__Bacteria; p__Actinobacteria; c__Actinobacteria; o__Actinomycetales; f__Nocardiaceae; g__Rhodococcus; s__ | 0 | 0 |
| k__Bacteria; p__Actinobacteria; c__Actinobacteria; o__Actinomycetales; f__Propionibacteriaceae; g__Propionibacterium; s__acnes | 0 | 0 |
| k__Bacteria; p__Bacteroidetes; c__Bacteroidia; o__Bacteroidales; f__[Paraprevotellaceae]; g__[Prevotella]; s__ | 0 | 5 |
| k__Bacteria; p__Bacteroidetes; c__Bacteroidia; o__Bacteroidales; f__Bacteroidaceae; g__Bacteroides; s__ | 0 | 0 |
| k__Bacteria; p__Bacteroidetes; c__Bacteroidia; o__Bacteroidales; f__Porphyromonadaceae; g__Porphyromonas; s__ | 0 | 0 |
| k__Bacteria; p__Bacteroidetes; c__Bacteroidia; o__Bacteroidales; f__Porphyromonadaceae; g__Porphyromonas; s__ | 0 | 0 |
| k__Bacteria; p__Bacteroidetes; c__Bacteroidia; o__Bacteroidales; f__Porphyromonadaceae; g__Porphyromonas; s__ | 0 | 0 |
| k__Bacteria; p__Bacteroidetes; c__Bacteroidia; o__Bacteroidales; f__Porphyromonadaceae; g__Porphyromonas; s__ | 0 | 0 |
| k__Bacteria; p__Bacteroidetes; c__Bacteroidia; o__Bacteroidales; f__Porphyromonadaceae; g__Porphyromonas; s__ | 0 | 0 |
| k__Bacteria; p__Bacteroidetes; c__Bacteroidia; o__Bacteroidales; f__Prevotellaceae; g__Prevotella; s__ | 0 | 0 |
| k__Bacteria; p__Bacteroidetes; c__Bacteroidia; o__Bacteroidales; f__Prevotellaceae; g__Prevotella; s__melaninogenica | 0 | 0 |
| Organism | A. | B. |
| k__Bacteria; p__Bacteroidetes; c__Bacteroidia; o__Bacteroidales; f__Prevotellaceae; g__Prevotella; s__nanceiensis | 0 | 0 |
| k__Bacteria; p__Bacteroidetes; c__Flavobacteriia; o__Flavobacteriales; f__[Weeksellaceae]; g__Wautersiella; s__ | 0 | 0 |
| k__Bacteria; p__Chloroflexi; c__Anaerolineae; o__SBR1031; f__A4b; g__; s__ | 1 | 0 |
| k__Bacteria; p__Cyanobacteria; c__Chloroplast; o__Streptophyta; f__; g__; s__ | 1 | 0 |
| k__Bacteria; p__Cyanobacteria; c__Oscillatoriophycideae; o__Chroococcales; f__Xenococcaceae; g__; s__ | 0 | 0 |
| k__Bacteria; p__Firmicutes; c__Bacilli; o__Bacillales; f__Staphylococcaceae; g__Jeotgalicoccus; s__ | 0 | 0 |
| k__Bacteria; p__Firmicutes; c__Bacilli; o__Bacillales; f__Staphylococcaceae; g__Staphylococcus; s__ | 0 | 0 |
| k__Bacteria; p__Firmicutes; c__Bacilli; o__Bacillales; f__Staphylococcaceae; g__Staphylococcus; s__ | 0 | 0 |
| k__Bacteria; p__Firmicutes; c__Bacilli; o__Bacillales; f__Staphylococcaceae; g__Staphylococcus; s__ | 0 | 5 |
| k__Bacteria; p__Firmicutes; c__Bacilli; o__Bacillales; f__Staphylococcaceae; g__Staphylococcus; s__aureus | 4857 | 11757 |
| k__Bacteria; p__Firmicutes; c__Bacilli; o__Bacillales; f__Staphylococcaceae; g__Staphylococcus; s__epidermidis | 0 | 0 |
| Organism | A. | B. |
| k__Bacteria; p__Firmicutes; c__Bacilli; o__Bacillales; f__Staphylococcaceae; g__Staphylococcus; s__epidermidis | 0 | 0 |
| k__Bacteria; p__Firmicutes; c__Bacilli; o__Bacillales; f__Staphylococcaceae; g__Staphylococcus; s__epidermidis | 0 | 1 |
| k__Bacteria; p__Firmicutes; c__Bacilli; o__Bacillales; f__Staphylococcaceae; g__Staphylococcus; s__equorum | 0 | 0 |
| k__Bacteria; p__Firmicutes; c__Bacilli; o__Bacillales; f__Staphylococcaceae; g__Staphylococcus; s__haemolyticus | 0 | 0 |
| k__Bacteria; p__Firmicutes; c__Bacilli; o__Gemellales; f__Gemellaceae; g__Gemella; s__ | 0 | 0 |
| k__Bacteria; p__Firmicutes; c__Bacilli; o__Lactobacillales; f__Aerococcaceae; g__; s__ | 0 | 0 |
| k__Bacteria; p__Firmicutes; c__Bacilli; o__Lactobacillales; f__Aerococcaceae; g__Aerococcus; s__ | 0 | 0 |
| k__Bacteria; p__Firmicutes; c__Bacilli; o__Lactobacillales; f__Aerococcaceae; g__Facklamia; s__ | 0 | 5 |
| k__Bacteria; p__Firmicutes; c__Bacilli; o__Lactobacillales; f__Enterococcaceae; g__Enterococcus; s__ | 0 | 0 |
| k__Bacteria; p__Firmicutes; c__Bacilli; o__Lactobacillales; f__Streptococcaceae; g__Streptococcus; s__ | 0 | 0 |
| k__Bacteria; p__Firmicutes; c__Bacilli; o__Lactobacillales; f__Streptococcaceae; g__Streptococcus; s__ | 0 | 0 |
| Organism | A. | B. |
| k__Bacteria; p__Firmicutes; c__Bacilli; o__Lactobacillales; f__Streptococcaceae; g__Streptococcus; s__ | 0 | 0 |
| k__Bacteria; p__Firmicutes; c__Bacilli; o__Lactobacillales; f__Streptococcaceae; g__Streptococcus; s__ | 0 | 0 |
| k__Bacteria; p__Firmicutes; c__Clostridia; o__Clostridiales; f__[Tissierellaceae]; g__Anaerococcus; s__ | 0 | 0 |
| k__Bacteria; p__Firmicutes; c__Clostridia; o__Clostridiales; f__[Tissierellaceae]; g__Anaerococcus; s__ | 0 | 0 |
| k__Bacteria; p__Firmicutes; c__Clostridia; o__Clostridiales; f__[Tissierellaceae]; g__Anaerococcus; s__ | 0 | 0 |
| k__Bacteria; p__Firmicutes; c__Clostridia; o__Clostridiales; f__[Tissierellaceae]; g__Anaerococcus; s__ | 0 | 0 |
| k__Bacteria; p__Firmicutes; c__Clostridia; o__Clostridiales; f__[Tissierellaceae]; g__Finegoldia; s__ | 0 | 0 |
| k__Bacteria; p__Firmicutes; c__Clostridia; o__Clostridiales; f__[Tissierellaceae]; g__Helcococcus; s__ | 0 | 0 |
| k__Bacteria; p__Firmicutes; c__Clostridia; o__Clostridiales; f__[Tissierellaceae]; g__Parvimonas; s__ | 0 | 0 |
| k__Bacteria; p__Firmicutes; c__Clostridia; o__Clostridiales; f__[Tissierellaceae]; g__Peptoniphilus; s__ | 0 | 0 |
| k__Bacteria; p__Firmicutes; c__Clostridia; o__Clostridiales; f__[Tissierellaceae]; g__Peptoniphilus; s__ | 0 | 0 |
| Organism | A. | B. |
| k__Bacteria; p__Firmicutes; c__Clostridia; o__Clostridiales; f__[Tissierellaceae]; g__Peptoniphilus; s__ | 0 | 0 |
| k__Bacteria; p__Firmicutes; c__Clostridia; o__Clostridiales; f__[Tissierellaceae]; g__Peptoniphilus; s__ | 0 | 0 |
| k__Bacteria; p__Firmicutes; c__Clostridia; o__Clostridiales; f__[Tissierellaceae]; g__Peptoniphilus; s__ | 0 | 0 |
| k__Bacteria; p__Firmicutes; c__Clostridia; o__Clostridiales; f__[Tissierellaceae]; g__Peptoniphilus; s__ | 0 | 0 |
| k__Bacteria; p__Firmicutes; c__Clostridia; o__Clostridiales; f__Clostridiaceae; g__Clostridium; s__ | 0 | 0 |
| k__Bacteria; p__Firmicutes; c__Clostridia; o__Clostridiales; f__Lachnospiraceae; g__Coprococcus; s__eutactus | 0 | 0 |
| k__Bacteria; p__Firmicutes; c__Clostridia; o__Clostridiales; f__Peptostreptococcaceae; g__Peptostreptococcus; s__anaerobius | 0 | 0 |
| k__Bacteria; p__Firmicutes; c__Clostridia; o__Clostridiales; f__Veillonellaceae; g__Pectinatus; s__ | 0 | 0 |
| k__Bacteria; p__Firmicutes; c__Erysipelotrichi; o__Erysipelotrichales; f__Erysipelotrichaceae; g__; s__ | 0 | 0 |
| k__Bacteria; p__Fusobacteria; c__Fusobacteriia; o__Fusobacteriales; f__Fusobacteriaceae; g__Fusobacterium; s__ | 0 | 0 |
| k__Bacteria; p__Fusobacteria; c__Fusobacteriia; o__Fusobacteriales; f__Fusobacteriaceae; g__Fusobacterium; s__ | 0 | 0 |
| Organism | A. | B. |
| k__Bacteria; p__Fusobacteria; c__Fusobacteriia; o__Fusobacteriales; f__Fusobacteriaceae; g__Fusobacterium; s__ | 0 | 0 |
| k__Bacteria; p__Fusobacteria; c__Fusobacteriia; o__Fusobacteriales; f__Fusobacteriaceae; g__Fusobacterium; s__ | 0 | 0 |
| k__Bacteria; p__Planctomycetes; c__Planctomycetia; o__Pirellulales; f__Pirellulaceae; g__; s__ | 5 | 0 |
| k__Bacteria; p__Proteobacteria; c__Alphaproteobacteria; o__Caulobacterales; f__Caulobacteraceae; g__Brevundimonas; s__diminuta | 0 | 4 |
| k__Bacteria; p__Proteobacteria; c__Alphaproteobacteria; o__Rhizobiales; f__; g__; s__ | 0 | 0 |
| k__Bacteria; p__Proteobacteria; c__Alphaproteobacteria; o__Rhizobiales; f__Methylobacteriaceae; g__; s__ | 0 | 0 |
| k__Bacteria; p__Proteobacteria; c__Alphaproteobacteria; o__Rhizobiales; f__Methylobacteriaceae; g__Methylobacterium; s__komagatae | 1 | 0 |
| k__Bacteria; p__Proteobacteria; c__Alphaproteobacteria; o__Sphingomonadales; f__Sphingomonadaceae; g__Sphingomonas; s__ | 0 | 0 |
| k__Bacteria; p__Proteobacteria; c__Betaproteobacteria; o__Burkholderiales; f__Alcaligenaceae; g__; s__ | 0 | 0 |
| k__Bacteria; p__Proteobacteria; c__Betaproteobacteria; o__Burkholderiales; f__Comamonadaceae; g__; s__ | 0 | 0 |
| k__Bacteria; p__Proteobacteria; c__Betaproteobacteria; o__Burkholderiales; f__Oxalobacteraceae; g__; s__ | 0 | 0 |
| Organism | A. | B. |
| k__Bacteria; p__Proteobacteria; c__Betaproteobacteria; o__Methylophilales; f__Methylophilaceae; g__; s__ | 0 | 0 |
| k__Bacteria; p__Proteobacteria; c__Betaproteobacteria; o__Neisseriales; f__Neisseriaceae; g__; s__ | 0 | 0 |
| k__Bacteria; p__Proteobacteria; c__Betaproteobacteria; o__Neisseriales; f__Neisseriaceae; g__Neisseria; s__ | 0 | 0 |
| k__Bacteria; p__Proteobacteria; c__Gammaproteobacteria; o__Alteromonadales; f__Shewanellaceae; g__Shewanella; s__algae | 0 | 0 |
| k__Bacteria; p__Proteobacteria; c__Gammaproteobacteria; o__Enterobacteriales; f__Enterobacteriaceae; g__; s__ | 0 | 0 |
| k__Bacteria; p__Proteobacteria; c__Gammaproteobacteria; o__Enterobacteriales; f__Enterobacteriaceae; g__; s__ | 0 | 0 |
| k__Bacteria; p__Proteobacteria; c__Gammaproteobacteria; o__Enterobacteriales; f__Enterobacteriaceae; g__; s__ | 0 | 0 |
| k__Bacteria; p__Proteobacteria; c__Gammaproteobacteria; o__Enterobacteriales; f__Enterobacteriaceae; g__; s__ | 0 | 0 |
| k__Bacteria; p__Proteobacteria; c__Gammaproteobacteria; o__Enterobacteriales; f__Enterobacteriaceae; g__; s__ | 0 | 0 |
| k__Bacteria; p__Proteobacteria; c__Gammaproteobacteria; o__Enterobacteriales; f__Enterobacteriaceae; g__; s__ | 0 | 0 |
| k__Bacteria; p__Proteobacteria; c__Gammaproteobacteria; o__Enterobacteriales; f__Enterobacteriaceae; g__; s__ | 0 | 0 |
| Organism | A. | B. |
| k__Bacteria; p__Proteobacteria; c__Gammaproteobacteria; o__Enterobacteriales; f__Enterobacteriaceae; g__; s__ | 0 | 0 |
| k__Bacteria; p__Proteobacteria; c__Gammaproteobacteria; o__Enterobacteriales; f__Enterobacteriaceae; g__; s__ | 0 | 0 |
| k__Bacteria; p__Proteobacteria; c__Gammaproteobacteria; o__Enterobacteriales; f__Enterobacteriaceae; g__Proteus; s__ | 0 | 1 |
| k__Bacteria; p__Proteobacteria; c__Gammaproteobacteria; o__Enterobacteriales; f__Enterobacteriaceae; g__Proteus; s__ | 0 | 0 |
| k__Bacteria; p__Proteobacteria; c__Gammaproteobacteria; o__Enterobacteriales; f__Enterobacteriaceae; g__Proteus; s__ | 0 | 3 |
| k__Bacteria; p__Proteobacteria; c__Gammaproteobacteria; o__Enterobacteriales; f__Enterobacteriaceae; g__Proteus; s__ | 0 | 0 |
| k__Bacteria; p__Proteobacteria; c__Gammaproteobacteria; o__Enterobacteriales; f__Enterobacteriaceae; g__Providencia; s__ | 0 | 0 |
| k__Bacteria; p__Proteobacteria; c__Gammaproteobacteria; o__Pasteurellales; f__Pasteurellaceae; g__Haemophilus; s__parainfluenzae | 0 | 0 |
| k__Bacteria; p__Proteobacteria; c__Gammaproteobacteria; o__Pasteurellales; f__Pasteurellaceae; g__Haemophilus; s__parainfluenzae | 0 | 0 |
| k__Bacteria; p__Proteobacteria; c__Gammaproteobacteria; o__Pasteurellales; f__Pasteurellaceae; g__Haemophilus; s__parainfluenzae | 0 | 0 |
| k__Bacteria; p__Proteobacteria; c__Gammaproteobacteria; o__Pasteurellales; f__Pasteurellaceae; g__Haemophilus; s__parainfluenzae | 0 | 0 |
| Organism | A. | B. |
| k__Bacteria; p__Proteobacteria; c__Gammaproteobacteria; o__Pasteurellales; f__Pasteurellaceae; g__Haemophilus; s__parainfluenzae | 0 | 0 |
| k__Bacteria; p__Proteobacteria; c__Gammaproteobacteria; o__Pseudomonadales; f__Moraxellaceae; g__; s__ | 0 | 0 |
| k__Bacteria; p__Proteobacteria; c__Gammaproteobacteria; o__Pseudomonadales; f__Moraxellaceae; g__Acinetobacter; s__ | 0 | 1 |
| k__Bacteria; p__Proteobacteria; c__Gammaproteobacteria; o__Pseudomonadales; f__Moraxellaceae; g__Acinetobacter; s__ | 0 | 0 |
| k__Bacteria; p__Proteobacteria; c__Gammaproteobacteria; o__Pseudomonadales; f__Moraxellaceae; g__Acinetobacter; s__ | 0 | 0 |
| k__Bacteria; p__Proteobacteria; c__Gammaproteobacteria; o__Pseudomonadales; f__Moraxellaceae; g__Acinetobacter; s__rhizosphaerae | 0 | 8 |
| k__Bacteria; p__Proteobacteria; c__Gammaproteobacteria; o__Pseudomonadales; f__Moraxellaceae; g__Acinetobacter; s__rhizosphaerae | 10208 | 2854 |
| k__Bacteria; p__Proteobacteria; c__Gammaproteobacteria; o__Pseudomonadales; f__Moraxellaceae; g__Enhydrobacter; s__ | 0 | 0 |
| k__Bacteria; p__Proteobacteria; c__Gammaproteobacteria; o__Pseudomonadales; f__Pseudomonadaceae; g__Pseudomonas; s__ | 0 | 0 |
| k__Bacteria; p__Proteobacteria; c__Gammaproteobacteria; o__Pseudomonadales; f__Pseudomonadaceae; g__Pseudomonas; s__ | 0 | 0 |
| k__Bacteria; p__Proteobacteria; c__Gammaproteobacteria; o__Pseudomonadales; f__Pseudomonadaceae; g__Pseudomonas; s__ | 2 | 0 |
| Organism | A. | B. |
| k__Bacteria; p__Proteobacteria; c__Gammaproteobacteria; o__Pseudomonadales; f__Pseudomonadaceae; g__Pseudomonas; s__ | 0 | 0 |
| k__Bacteria; p__Proteobacteria; c__Gammaproteobacteria; o__Pseudomonadales; f__Pseudomonadaceae; g__Pseudomonas; s__ | 0 | 0 |
| k__Bacteria; p__Proteobacteria; c__Gammaproteobacteria; o__Pseudomonadales; f__Pseudomonadaceae; g__Pseudomonas; s__ | 0 | 0 |
| k__Bacteria; p__Proteobacteria; c__Gammaproteobacteria; o__Pseudomonadales; f__Pseudomonadaceae; g__Pseudomonas; s__stutzeri | 0 | 0 |
| Total Reads | 15075 | 14645 |

A. Number of reads not *S. aureus* or *A. rhizosphaerae* = 10/15075

B. Number of reads not *S. aureus* or *A. rhizosphaerae* = 20/14645

No evidence of extraction reagent contamination

**Supplementary Table S8 Additional File 5**

**Positive control data (swabs): A***. Pseudomonas aeruginosa* ATCC 27856 swab DNA extraction method **B**. *Staphylococcus aureus* ATCC 29213 swab DNA extraction method

| OTU reference no. | Top Blast Hit | A. | B. |
| --- | --- | --- | --- |
| AF084237 | Eukaryota; __Opisthokonta; __Metazoa; __Porifera; __Demospongiae; __'Prosuberites'; __'Prosuberites'_laughlini | 3 | 1 |
| 4466258 | k__Archaea; p__Crenarchaeota; c__Thaumarchaeota; o__Cenarchaeales; f__Cenarchaeaceae; g__Cenarchaeum; s__symbiosum | 0 | 2 |
| 1047077 | k__Bacteria; p__Actinobacteria; c__Actinobacteria; o__Actinomycetales; f__Actinomycetaceae; g__Actinomyces; s__ | 0 | 0 |
| 947112 | k__Bacteria; p__Actinobacteria; c__Actinobacteria; o__Actinomycetales; f__Corynebacteriaceae; g__Corynebacterium; s__ | 0 | 0 |
| 1012948 | k__Bacteria; p__Actinobacteria; c__Actinobacteria; o__Actinomycetales; f__Corynebacteriaceae; g__Corynebacterium; s__ | 1 | 0 |
| 420127 | k__Bacteria; p__Actinobacteria; c__Actinobacteria; o__Actinomycetales; f__Corynebacteriaceae; g__Corynebacterium; s__ | 0 | 0 |
| 109591 | k__Bacteria; p__Actinobacteria; c__Actinobacteria; o__Actinomycetales; f__Dermabacteraceae; g__Dermabacter; s__ | 0 | 0 |

| OTU reference no. | Top blast hit | A. | B. |
| --- | --- | --- | --- |
| 4411138 | k__Bacteria; p__Actinobacteria; c__Actinobacteria; o__Actinomycetales; f__Micrococcaceae; g__Rothia; s__mucilaginosa | 3. | 0 |
| 1106384 | k__Bacteria; p__Actinobacteria; c__Coriobacteriia; o__Coriobacteriales; f__Coriobacteriaceae; g__; s__ | 0 | 0 |
| 471157 | k__Bacteria; p__Actinobacteria; c__Coriobacteriia; o__Coriobacteriales; f__Coriobacteriaceae; g__Slackia; s__ | 0 | 0 |
| New.CleanUp.ReferenceOTU1053 | k__Bacteria; p__Bacteroidetes; c__Bacteroidia; o__Bacteroidales; f__Bacteroidaceae; g__Bacteroides; s__ | 0 | 1 |
| 4439360 | k__Bacteria; p__Bacteroidetes; c__Bacteroidia; o__Bacteroidales; f__Bacteroidaceae; g__Bacteroides; s__ | 0 | 0 |
| 3600504 | k__Bacteria; p__Bacteroidetes; c__Bacteroidia; o__Bacteroidales; f__Bacteroidaceae; g__Bacteroides; s__ | 0 | 1 |
| 4433947 | k__Bacteria; p__Bacteroidetes; c__Bacteroidia; o__Bacteroidales; f__Bacteroidaceae; g__Bacteroides; s__ | 0 | 0 |
| 979707 | k__Bacteria; p__Bacteroidetes; c__Bacteroidia; o__Bacteroidales; f__Porphyromonadaceae; g__Porphyromonas; s__ | 1 | 0 |
| New.ReferenceOTU7 | k__Bacteria; p__Bacteroidetes; c__Bacteroidia; o__Bacteroidales; f__Porphyromonadaceae; g__Porphyromonas; s__ | 0 | 0 |

| OTU reference no. | Top Blast Hit | A. | B. |
| --- | --- | --- | --- |
| 692756 | k__Bacteria; p__Bacteroidetes; c__Bacteroidia; o__Bacteroidales; f__Porphyromonadaceae; g__Porphyromonas; s__ | 0 | 0 |
| 495451 | k__Bacteria; p__Bacteroidetes; c__Bacteroidia; o__Bacteroidales; f__Porphyromonadaceae; g__Porphyromonas; s__ | 1 | 4 |
| 495017 | k__Bacteria; p__Bacteroidetes; c__Bacteroidia; o__Bacteroidales; f__Porphyromonadaceae; g__Porphyromonas; s__ | 0 | 0 |
| 760967 | k__Bacteria; p__Bacteroidetes; c__Bacteroidia; o__Bacteroidales; f__Prevotellaceae; g__Prevotella; s__ | 1 | 5 |
| New.ReferenceOTU15 | k__Bacteria; p__Bacteroidetes; c__Bacteroidia; o__Bacteroidales; f__Prevotellaceae; g__Prevotella; s__ | 0 | 0 |
| 4295230 | k__Bacteria; p__Bacteroidetes; c__Flavobacteriia; o__Flavobacteriales; f__[Weeksellaceae]; g__Elizabethkingia; s__meningoseptica | 3 | 2 |
| 4420570 | k__Bacteria; p__Cyanobacteria; c__Chloroplast; o__Streptophyta; f__; g__; s__ | 2 | 5 |
| 3385021 | k__Bacteria; p__Firmicutes; c__Bacilli; o__Bacillales; f__Staphylococcaceae; g__Staphylococcus; s__ | 0 | 366 |
| 1040220 | k__Bacteria; p__Firmicutes; c__Bacilli; o__Bacillales; f__Staphylococcaceae; g__Staphylococcus; s__aureus | 0 | 375 |
| 4345285 | k__Bacteria; p__Firmicutes; c__Bacilli; o__Bacillales; f__Staphylococcaceae; g__Staphylococcus; s__aureus | 8 | 25975 |
| 2896107 | k__Bacteria; p__Firmicutes; c__Bacilli; o__Bacillales; f__Staphylococcaceae; g__Staphylococcus; s__aureus | 0 | 232 |
| OTU reference no. | Top Blast Hit | A. | B. |
| 928538 | k__Bacteria; p__Firmicutes; c__Bacilli; o__Bacillales; f__Staphylococcaceae; g__Staphylococcus; s__aureus | 2 | 132 |
| 4312969 | k__Bacteria; p__Firmicutes; c__Bacilli; o__Bacillales; f__Staphylococcaceae; g__Staphylococcus; s__epidermidis | 0 | 638 |
| 4446902 | k__Bacteria; p__Firmicutes; c__Bacilli; o__Gemellales; f__Gemellaceae; g__Gemella; s__ | 0 | 2 |
| 1033413 | k__Bacteria; p__Firmicutes; c__Bacilli; o__Lactobacillales; f__Enterococcaceae; g__Enterococcus; s__ | 0 | 0 |
| 4316928 | k__Bacteria; p__Firmicutes; c__Bacilli; o__Lactobacillales; f__Enterococcaceae; g__Enterococcus; s__ | 0 | 0 |
| 4425214 | k__Bacteria; p__Firmicutes; c__Bacilli; o__Lactobacillales; f__Streptococcaceae; g__Streptococcus; s__ | 0 | 0 |
| 14344207 | k__Bacteria; p__Firmicutes; c__Bacilli; o__Lactobacillales; f__Streptococcaceae; g__Streptococcus; s__anginosus | 1 | 0 |
| 4309301 | k__Bacteria; p__Firmicutes; c__Bacilli; o__Lactobacillales; f__Streptococcaceae; g__Streptococcus; s__infantis | 14 | 15 |
| 4335578 | k__Bacteria; p__Firmicutes; c__Clostridia; o__Clostridiales; f__[Mogibacteriaceae]; g__; s__ | 0 | 0 |
| 1820843 | k__Bacteria; p__Firmicutes; c__Clostridia; o__Clostridiales; f__[Mogibacteriaceae]; g__Mogibacterium; s__ | 0 | 0 |

| OTU reference no. | Top Blast Hit | A. | B. |
| --- | --- | --- | --- |
| 1334786 | k__Bacteria; p__Firmicutes; c__Clostridia; o__Clostridiales; f__[Tissierellaceae]; g__Anaerococcus; s__ | 0 | 0 |
| 4224038 | k__Bacteria; p__Firmicutes; c__Clostridia; o__Clostridiales; f__[Tissierellaceae]; g__Anaerococcus; s__ | 0 | 0 |
| 5030372 | k__Bacteria; p__Firmicutes; c__Clostridia; o__Clostridiales; f__[Tissierellaceae]; g__Anaerococcus; s__ | 0 | 0 |
| 44763950 | k__Bacteria; p__Firmicutes; c__Clostridia; o__Clostridiales; f__[Tissierellaceae]; g__Anaerococcus; s__ | 1 | 1 |
| 30062 | k__Bacteria; p__Firmicutes; c__Clostridia; o__Clostridiales; f__[Tissierellaceae]; g__Anaerococcus; s__ | 0 | 0 |
| 4349519 | k__Bacteria; p__Firmicutes; c__Clostridia; o__Clostridiales; f__[Tissierellaceae]; g__Anaerococcus; s__ | 0 | 0 |
| New.CleanUp.ReferenceOTU188 | k__Bacteria; p__Firmicutes; c__Clostridia; o__Clostridiales; f__[Tissierellaceae]; g__Anaerococcus; s__ | 0 | 0 |
| 14288 | k__Bacteria; p__Firmicutes; c__Clostridia; o__Clostridiales; f__[Tissierellaceae]; g__Anaerococcus; s__ | 0 | 0 |
| New.CleanUp.ReferenceOTU1134 | k__Bacteria; p__Firmicutes; c__Clostridia; o__Clostridiales; f__[Tissierellaceae]; g__Anaerococcus; s__ | 0 | 0 |
| 224222 | k__Bacteria; p__Firmicutes; c__Clostridia; o__Clostridiales; f__[Tissierellaceae]; g__Anaerococcus; s__ | 0 | 0 |

| OTU reference no. | Top Blast Hit | A. | B. |  |
| --- | --- | --- | --- | --- |
| 968363 | k__Bacteria; p__Firmicutes; c__Clostridia; o__Clostridiales; f__[Tissierellaceae]; g__Anaerococcus; s__ | 0 | 0 |  |
| 1096610 | k__Bacteria; p__Firmicutes; c__Clostridia; o__Clostridiales; f__[Tissierellaceae]; g__Finegoldia; s__ | 1 | 0 |  |
| New.CleanUp.ReferenceOTU341 | k__Bacteria; p__Firmicutes; c__Clostridia; o__Clostridiales; f__[Tissierellaceae]; g__Finegoldia; s__ | 0 | 0 |  |
| 793524 | k__Bacteria; p__Firmicutes; c__Clostridia; o__Clostridiales; f__[Tissierellaceae]; g__Helcococcus; s__ | 0 | 0 |  |
| 325610 | k__Bacteria; p__Firmicutes; c__Clostridia; o__Clostridiales; f__[Tissierellaceae]; g__Helcococcus; s__ | 0 | 0 |  |
| 851704 | k__Bacteria; p__Firmicutes; c__Clostridia; o__Clostridiales; f__[Tissierellaceae]; g__Parvimonas; s__ | 0 | 1 |  |
| 4429335 | k__Bacteria; p__Firmicutes; c__Clostridia; o__Clostridiales; f__[Tissierellaceae]; g__Peptoniphilus; s__ | 1 | 0 |  |
| 4397098 | k__Bacteria; p__Firmicutes; c__Clostridia; o__Clostridiales; f__[Tissierellaceae]; g__Peptoniphilus; s__ | 0 | 0 |  |
| 494906 | k__Bacteria; p__Firmicutes; c__Clostridia; o__Clostridiales; f__[Tissierellaceae]; g__Peptoniphilus; s__ | 0 | 0 |  |
| 1019823 | k__Bacteria; p__Firmicutes; c__Clostridia; o__Clostridiales; f__[Tissierellaceae]; g__Peptoniphilus; s__ | 0 | 0 |  |
| 654307 | k__Bacteria; p__Firmicutes; c__Clostridia; o__Clostridiales; f__[Tissierellaceae]; g__Peptoniphilus; s__ | 0 | 0 |  |
| OTU reference no. | Top Blast Hit | A. | B. |  |
| 4344651 | k__Bacteria; p__Firmicutes; c__Clostridia; o__Clostridiales; f__[Tissierellaceae]; g__Peptoniphilus; s__ | 1 | 0 |  |
| 527630 | k__Bacteria; p__Firmicutes; c__Clostridia; o__Clostridiales; f__Peptostreptococcaceae; g__Peptostreptococcus; s__ | 0 | 1 | |
| New.ReferenceOTU8 | k__Bacteria; p__Firmicutes; c__Clostridia; o__Clostridiales; f__Ruminococcaceae; g__; s__ | 14 | 11 |  |
| 4425172 | k__Bacteria; p__Firmicutes; c__Clostridia; o__Clostridiales; f__Veillonellaceae; g__Selenomonas; s__ | 0 | 0 |  |
| 851938 | k__Bacteria; p__Firmicutes; c__Erysipelotrichi; o__Erysipelotrichales; f__Erysipelotrichaceae; g__Bulleidia; | 0 | 0 |  |
| 4323555 | k__Bacteria; p__Fusobacteria; c__Fusobacteriia; o__Fusobacteriales; f__Fusobacteriaceae; g__Fusobacterium; s__ | 0 | 0 |  |
| 4319899 | k__Bacteria; p__Fusobacteria; c__Fusobacteriia; o__Fusobacteriales; f__Fusobacteriaceae; g__Fusobacterium; s__ | 0 | 0 |  |
| 4396717 | k__Bacteria; p__Proteobacteria; c__Alphaproteobacteria; o__Rhizobiales; f__Methylobacteriaceae; g__Methylobacterium; s__hispanicum | 0 | 0 |  |
| 4245392 | k__Bacteria; p__Proteobacteria; c__Alphaproteobacteria; o__Rhodobacterales; f__Rhodobacteraceae; g__Paracoccus; s__aminovorans | 0 | 0 |  |

| OTU reference no. | | Top Blast Hit | | A. | B. | |  |
| --- | --- | --- | --- | --- | --- | --- | --- |
| 4376318 | | k__Bacteria; p__Proteobacteria; c__Alphaproteobacteria; o__Rhodobacterales; f__Rhodobacteraceae; g__Paracoccus; s__marcusii | | 0 | 0 | |  |
| 2693224 | | k__Bacteria; p__Proteobacteria; c__Alphaproteobacteria; o__Sphingomonadales; f__Sphingomonadaceae; g__Sphingomonas; s__ | | 0 | 0 | |  |
| 4396235 | | k__Bacteria; p__Proteobacteria; c__Betaproteobacteria; o__Neisseriales; f__Neisseriaceae; g__Neisseria; s__ | | 0 | 1 | |  |
| 4429754 | | k__Bacteria; p__Proteobacteria; c__Deltaproteobacteria; o__NB1-j; f__NB1-i; g__; s__ | | 11 | 3 | |  |
| 4456435 | | k__Bacteria; p__Proteobacteria; c__Epsilonproteobacteria; o__Campylobacterales; f__Campylobacteraceae; g__Arcobacter; s__ | | 0 | 4 | |  |
| 143462 | | k__Bacteria; p__Proteobacteria; c__Gammaproteobacteria; o__Alteromonadales; f__[Chromatiaceae]; g__; s__ | | 1 | 0 | |  |
| 263243 | | k__Bacteria; p__Proteobacteria; c__Gammaproteobacteria; o__Chromatiales; f__; g__; s__ | | 1 | 0 | |  |
| 91557 | | k__Bacteria; p__Proteobacteria; c__Gammaproteobacteria; o__Enterobacteriales; f__Enterobacteriaceae; g__; s__ | | 0 | 0 | |  |
| 668514 | | k__Bacteria; p__Proteobacteria; c__Gammaproteobacteria; o__Enterobacteriales; f__Enterobacteriaceae; g__; s__ | | 0 | 0 | |  |
| OTU reference no. | Top Blast Hit | | A. | | B. | | |
| 816702 | k__Bacteria; p__Proteobacteria; c__Gammaproteobacteria; o__Enterobacteriales; f__Enterobacteriaceae; g__Klebsiella; s__ | | 0 | | 0 | | |
| 4363066 | k__Bacteria; p__Proteobacteria; c__Gammaproteobacteria; o__Pasteurellales; f__Pasteurellaceae; g__Aggregatibacter; s__ | | 0 | | 0 | | |
| 4477696 | k__Bacteria; p__Proteobacteria; c__Gammaproteobacteria; o__Pasteurellales; f__Pasteurellaceae; g__Haemophilus; s__parainfluenzae | | 0 | | 1 | | |
| 818602 | k__Bacteria; p__Proteobacteria; c__Gammaproteobacteria; o__Pseudomonadales; f__Pseudomonadaceae; g__Pseudomonas; s__ | | 3 | | 16 | | |
| 4327501 | k__Bacteria; p__Proteobacteria; c__Gammaproteobacteria; o__Pseudomonadales; f__Pseudomonadaceae; g__Pseudomonas; s__ | | 0 | | 0 | | |
| 242070 | k__Bacteria; p__Proteobacteria; c__Gammaproteobacteria; o__Pseudomonadales; f__Pseudomonadaceae; g__Pseudomonas; s__ | | 591 | | 0 | | |
| 133961 | k__Bacteria; p__Proteobacteria; c__Gammaproteobacteria; o__Pseudomonadales; f__Pseudomonadaceae; g__Pseudomonas; s__ | | 0 | | 1 | | |
| 225088 | k__Bacteria; p__Proteobacteria; c__Gammaproteobacteria; o__Pseudomonadales; f__Pseudomonadaceae; g__Pseudomonas; s__ | | 39265 | | 1 | | |
| 4327028 | k__Bacteria; p__Proteobacteria; c__Gammaproteobacteria; o__Pseudomonadales; f__Pseudomonadaceae; g__Pseudomonas; s__ | | 0 | | 0 | | |
| 4436550 | k__Bacteria; p__Proteobacteria; c__Gammaproteobacteria; o__Thiohalorhabdales; f__; g__; s__ | | 1 | | | 2 | |
|  | **Total no. of reads** | | **39931** | | **27799** | | |

A. Number of reads not *Pseudomonas* spp**.: 72 /39931** B. Number of reads not *Staphylococcus* spp**.: 81/27799**

Although the control bacteria were from an American type culture collection (ATCC) these organism had been subcultured in a clinical laboratory many times and the purity of the *S.aureus* control may have been compromised. Therefore *Staphylococcus* spp. and *S. epidermidis* have been included as control results.

Results do not indicate evidence of reagent contamination.

**Supplementary Table S9 Additional File 5**

**Negative control data (swabs)**:

A) Unused Copan swab sample using swab DNA extraction method to determine if any significant swab contamination

B) Reagents only (No sample) using swab DNA extraction method to determine if any significant reagent contamination

| OTU reference no. | | Top Blast Hit | | A. | | B. | |  |
| --- | --- | --- | --- | --- | --- | --- | --- | --- |
| 12574 | | k__Bacteria; p__Actinobacteria; c__Actinobacteria; o__Actinomycetales; f__Actinomycetaceae; g__Actinomyces; s__ | | 50 | | 2 | |  |
| 866280 | | k__Bacteria; p__Actinobacteria; c__Actinobacteria; o__Actinomycetales; f__Micrococcaceae; g__Rothia; s__mucilaginosa | | 39 | | 0 | |  |
| 1106384 | | k__Bacteria; p__Actinobacteria; c__Coriobacteriia; o__Coriobacteriales; f__Coriobacteriaceae; g__; s__ | | 6 | | 0 | |  |
| 4433947 | | k__Bacteria; p__Bacteroidetes; c__Bacteroidia; o__Bacteroidales; f__Bacteroidaceae; g__Bacteroides; s__ | | 2 | | 0 | |  |
| New.CleanUp.ReferenceOTU42 | | k__Bacteria; p__Bacteroidetes; c__Bacteroidia; o__Bacteroidales; f__Bacteroidaceae; g__Bacteroides; s__ | | 0 | | 0 | |  |
| 3211875 | | k__Bacteria; p__Bacteroidetes; c__Bacteroidia; o__Bacteroidales; f__Bacteroidaceae; g__Bacteroides; s__ | | 10 | | 0 | |  |
| New.CleanUp.ReferenceOTU194 | | k__Bacteria; p__Bacteroidetes; c__Bacteroidia; o__Bacteroidales; f__Bacteroidaceae; g__Bacteroides; s__ | | 0 | | 0 | |  |
| OTU reference no. | | Top Blast Hit | | A. | | B. | |  |
| New.CleanUp.ReferenceOTU77 | | k__Bacteria; p__Bacteroidetes; c__Bacteroidia; o__Bacteroidales; f__Bacteroidaceae; g__Bacteroides; s__ | | 0 | | 0 | |  |
| 4439360 | | k__Bacteria; p__Bacteroidetes; c__Bacteroidia; o__Bacteroidales; f__Bacteroidaceae; g__Bacteroides; s__ | | 183 | | 0 | |  |
| New.CleanUp.ReferenceOTU216 | | k__Bacteria; p__Bacteroidetes; c__Bacteroidia; o__Bacteroidales; f__Bacteroidaceae; g__Bacteroides; s__ | | 0 | | 0 | |  |
| 3600504 | | k__Bacteria; p__Bacteroidetes; c__Bacteroidia; o__Bacteroidales; f__Bacteroidaceae; g__Bacteroides; s__ | | 20 | | 8 | |  |
| **495451** | | **k__Bacteria; p__Bacteroidetes; c__Bacteroidia; o__Bacteroidales; f__Porphyromonadaceae; g__Porphyromonas; s__** | | **57** | | **4** | |  |
| 979707 | | k__Bacteria; p__Bacteroidetes; c__Bacteroidia; o__Bacteroidales; f__Porphyromonadaceae; g__Porphyromonas; s__ | | 0 | | 0 | |  |
| 4321559 | | k__Bacteria; p__Bacteroidetes; c__Bacteroidia; o__Bacteroidales; f__Porphyromonadaceae; g__Porphyromonas; s__ | | 29 | | 2 | |  |
| New.CleanUp.ReferenceOTU246 | | k__Bacteria; p__Bacteroidetes; c__Bacteroidia; o__Bacteroidales; f__Prevotellaceae; g__Prevotella; s__ | | 0 | | 0 | |  |
| New.CleanUp.ReferenceOTU46 | | k__Bacteria; p__Bacteroidetes; c__Bacteroidia; o__Bacteroidales; f__Prevotellaceae; g__Prevotella; s__ | | 0 | | 0 | |  |
| New.CleanUp.ReferenceOTU55 | | k__Bacteria; p__Bacteroidetes; c__Bacteroidia; o__Bacteroidales; f__Prevotellaceae; g__Prevotella; s__ | | 1 | | 0 | |  |
| New.CleanUp.ReferenceOTU112 | | k__Bacteria; p__Bacteroidetes; c__Bacteroidia; o__Bacteroidales; f__Prevotellaceae; g__Prevotella; s__ | | 0 | | 0 | |  |
| OTU Reference no. | | Top Blast Hit | | A. | | B. | | |
| **760967** | | **k__Bacteria; p__Bacteroidetes; c__Bacteroidia; o__Bacteroidales; f__Prevotellaceae; g__Prevotella; s__** | | **973** | | **0** | | |
| **4345285** | | **k__Bacteria; p__Firmicutes; c__Bacilli; o__Bacillales; f__Staphylococcaceae; g__Staphylococcus; s__aureus** | | **69** | | **6** | | |
| **1033413** | | **k__Bacteria; p__Firmicutes; c__Bacilli; o__Lactobacillales; f__Enterococcaceae; g__Enterococcus; s__** | | **47** | | **0** | | |
| New.CleanUp.ReferenceOTU265 | | k__Bacteria; p__Firmicutes; c__Bacilli; o__Lactobacillales; f__Enterococcaceae; g__Enterococcus; s__haemoperoxidus | | 0 | | 0 | | |
| 4425214 | | k__Bacteria; p__Firmicutes; c__Bacilli; o__Lactobacillales; f__Streptococcaceae; g__Streptococcus; s__ | | 87 | | 0 | | |
| 4442130 | | k__Bacteria; p__Firmicutes; c__Bacilli; o__Lactobacillales; f__Streptococcaceae; g__Streptococcus; s__ | | 21 | | 0 | | |
| 4344207 | | k__Bacteria; p__Firmicutes; c__Bacilli; o__Lactobacillales; f__Streptococcaceae; g__Streptococcus; s__anginosus | | 0 | | 0 | | |
| **4309301** | | **k__Bacteria; p__Firmicutes; c__Bacilli; o__Lactobacillales; f__Streptococcaceae; g__Streptococcus; s__infantis** | | **193** | | **25** | | |
| 4335578 | | k__Bacteria; p__Firmicutes; c__Clostridia; o__Clostridiales; f__[Mogibacteriaceae]; g__; s__ | | 6 | | 0 | | |
| 820843 | | k__Bacteria; p__Firmicutes; c__Clostridia; o__Clostridiales; f__[Mogibacteriaceae]; g__Mogibacterium; s__ | | 10 | | 0 | | |
| **4476950** | | **k__Bacteria; p__Firmicutes; c__Clostridia; o__Clostridiales; f__[Tissierellaceae]; g__Anaerococcus; s__** | | **68** | | **3** | | |
| OTU reference no. | | Top Blast Hit | | A. | | B. | | |
| 968363 | | k__Bacteria; p__Firmicutes; c__Clostridia; o__Clostridiales; f__[Tissierellaceae]; g__Anaerococcus; s__ | | 0 | | 0 | | |
| **1096610** | | **k__Bacteria; p__Firmicutes; c__Clostridia; o__Clostridiales; f__[Tissierellaceae]; g__Finegoldia; s__** | | **41** | | **0** | | |
| 851704 | | k__Bacteria; p__Firmicutes; c__Clostridia; o__Clostridiales; f__[Tissierellaceae]; g__Parvimonas; s__ | | 3 | | 0 | | |
| **4429335** | | **k__Bacteria; p__Firmicutes; c__Clostridia; o__Clostridiales; f__[Tissierellaceae]; g__Peptoniphilus; s__** | | **31** | | **0** | | |
| 1007750 | | k__Bacteria; p__Firmicutes; c__Clostridia; o__Clostridiales; f__[Tissierellaceae]; g__Peptoniphilus; s__ | | 0 | | 0 | | |
| New.CleanUp.ReferenceOTU20 | | k__Bacteria; p__Firmicutes; c__Clostridia; o__Clostridiales; f__Lachnospiraceae; g__Moryella; s__ | | 26 | | 0 | | |
| 527630 | | k__Bacteria; p__Firmicutes; c__Clostridia; o__Clostridiales; f__Peptostreptococcaceae; g__Peptostreptococcus; s__ | | 26 | | 0 | | |
| 851938 | | k__Bacteria; p__Firmicutes; c__Erysipelotrichi; o__Erysipelotrichales; f__Erysipelotrichaceae; g__Bulleidia; s__moorei | | 0 | | 0 | | |
| 4323555 | | k__Bacteria; p__Fusobacteria; c__Fusobacteriia; o__Fusobacteriales; f__Fusobacteriaceae; g__Fusobacterium; s__ | | 0 | | 0 | | |
| 68617 | | k__Bacteria; p__Proteobacteria; c__Betaproteobacteria; o__Burkholderiales; f__Alcaligenaceae; g__Achromobacter; s__ | | 26 | | 3 | | |
| 4469492 | | k__Bacteria; p__Proteobacteria; c__Betaproteobacteria; o__Burkholderiales; f__Comamonadaceae; g__Delftia; s__ | | 38 | | 0 | | |
| OTU reference no. | Top Blast Hit | | A. | | B. | |  |  |
| 136025 | k__Bacteria; p__Proteobacteria; c__Betaproteobacteria; o__Burkholderiales; f__Oxalobacteraceae; g__Ralstonia; s__ | | 18 | | 12 | |  |  |
| 4396235 | k__Bacteria; p__Proteobacteria; c__Betaproteobacteria; o__Neisseriales; f__Neisseriaceae; g__Neisseria; s__ | | 55 | | 0 | |  |  |
| 4429754 | k__Bacteria; p__Proteobacteria; c__Deltaproteobacteria; o__NB1-j; f__NB1-i; g__; s__ | | 4 | | 6 | |  |  |
| 263243 | k__Bacteria; p__Proteobacteria; c__Gammaproteobacteria; o__Chromatiales; f__; g__; s__ | | 21 | | 12 | |  |  |
| New.CleanUp.ReferenceOTU40 | k__Bacteria; p__Proteobacteria; c__Gammaproteobacteria; o__Enterobacteriales; f__Enterobacteriaceae; g__; s__ | | 0 | | 0 | |  |  |
| 67813 | k__Bacteria; p__Proteobacteria; c__Gammaproteobacteria; o__Enterobacteriales; f__Enterobacteriaceae; g__; s__ | | 0 | | 0 | |  |  |
| 4456705 | k__Bacteria; p__Proteobacteria; c__Gammaproteobacteria; o__HTCC2188; f__HTCC2089; g__; s__ | | 28 | | 0 | |  |  |
| 4477696 | k__Bacteria; p__Proteobacteria; c__Gammaproteobacteria; o__Pasteurellales; f__Pasteurellaceae; g__Haemophilus; s__parainfluenzae | | 69 | | 0 | |  |  |
| 4364813 | k__Bacteria; p__Proteobacteria; c__Gammaproteobacteria; o__Pseudomonadales; f__Pseudomonadaceae; g__; s__ | | 36 | | J | |  |  |
| 133961 | k__Bacteria; p__Proteobacteria; c__Gammaproteobacteria; o__Pseudomonadales; f__Pseudomonadaceae; g__; s__ | | 15 | | 0 | |  |  |
| **225088** | **k__Bacteria; p__Proteobacteria; c__Gammaproteobacteria; o__Pseudomonadales; f__Pseudomonadaceae; g__Pseudomonas; s__** | | **29** | | **11** | |  |  |
| OTU reference no. | | Top Blast Hit | | A. | | B. | | |
| 4327028 | | k__Bacteria; p__Proteobacteria; c__Gammaproteobacteria; o__Pseudomonadales; f__Pseudomonadaceae; g__Pseudomonas; s__ | | 95 | | 1 | | |
| New.CleanUp.ReferenceOTU288 | | k__Bacteria; p__Proteobacteria; c__Gammaproteobacteria; o__Pseudomonadales; f__Pseudomonadaceae; g__Pseudomonas; s__ | | 0 | | 0 | | |
| 818602 | | k__Bacteria; p__Proteobacteria; c__Gammaproteobacteria; o__Pseudomonadales; f__Pseudomonadaceae; g__Pseudomonas; s__ | | 5396 | | 23 | | |
| New.CleanUp.ReferenceOTU141 | | k__Bacteria; p__Proteobacteria; c__Gammaproteobacteria; o__Pseudomonadales; f__Pseudomonadaceae; g__Pseudomonas; s__ | | 0 | | 0 | | |
| New.CleanUp.ReferenceOTU244 | | k__Bacteria; p__Proteobacteria; c__Gammaproteobacteria; o__Pseudomonadales; f__Pseudomonadaceae; g__Pseudomonas; s__ | | 0 | | 0 | | |
| 4436550 | | k__Bacteria; p__Proteobacteria; c__Gammaproteobacteria; o__Thiohalorhabdales; f__; g__; s__ | | 32 | | 6 | | |
| New.CleanUp.ReferenceOTU127 | | k__Bacteria; p__Tenericutes; c__Mollicutes; o__RF39; f__; g__; s__ | | 4 | | 10 | | |
| **Total No. of reads** | |  | | **7864** | | **134** | | |

**Note:** Reference identification numbers in red (above) were identified from the negative Copan swab data and matched with the same reference identification number from the data (indicated in Supplementary Table S10 below) generated from Copan swab ulcer samples used in this study. Swab reads do not appear to be biased relative to tissue biopsies due to use of Copan swabs for sample collection.

| **Reference OTU no.** | **Copan negative swab control Total no. reads** | **Copan Swab sample data**  **Total no. reads** | **Tissue biopsy sample data**  **Total no. reads** |
| --- | --- | --- | --- |
| 495451 | 57 | 5138 | 547 |
| 760967 | 973 | 1199 | 7309 |
| 4345285 | 69 | 91101 | 2673 |
| 1033413 | 47 | 2273 | 11223 |
| 4309301 | 193 | 650 | 3634 |
| 4476950 | 68 | 416 | 152 |
| 1096610 | 41 | 13275 | 10540 |
| 4429335 | 31 | 402 | 0 |
| 225088 | 29 | 16500 | 33657 |

**Supplementary Table S 10 Additional File 5**

**Copan swab control data**

**Supplementary Table S 11 Additional File 5**

**Quantitative PCR data for positive and negative swab controls**

| Well Name | Well Type | Threshold (dR) | Ct (dR) | Quantity (copies) | Background corrected  (-524.6) |
| --- | --- | --- | --- | --- | --- |
| 16S | Standard | 3544.042 | 16.04 | 1000000 |  |
| 16S | Standard | 3544.042 | 19.42 | 100000 |  |
| 16S | Standard | 3544.042 | 19.31 | 100000 |  |
| 16S | Standard | 3544.042 | 23.7 | 10000 |  |
| 16S | Standard | 3544.042 | 24.1 | 10000 |  |
| 16S | Standard | 3544.042 | 27.68 | 1000 |  |
| 16S | Standard | 3544.042 | 28.9 | 1000 |  |
| Copan swab no sample swab DNA extraction method | Unknown | 3544.042 | 25.71 | 3791 | 3266.4 |
| H_2_O negative QPCR control | Unknown | 3544.042 | 29.32 | 524.6 | Zero copies |
| No sample swab DNA extraction method | Unknown | 3544.042 | 28.11 | 1020 | 495.4 |
| No sample swab DNA extraction method | Unknown | 3544.042 | 28.43 | 854.3 | 329.7 |
| Pseudo control. swab DNA extraction method | Unknown | 3544.042 | 16.8 | 498000 | 497476 |
| Staph control .swab DNA extraction method | Unknown | 3544.042 | 15.94 | 796900 | 796376 |
